# Supplementary material for: Evaluating vaccine allocation strategies using simulation-assisted causal modeling
Source: Patterns (N Y). 2023 May 3;4(6):100739. doi: 10.1016/j.patter.2023.100739 (PMC10155501; doi:10.1016/j.patter.2023.100739)
Supplement: Document S1. Supplemental experimental procedures and Figures S1–S5 [file mmc1.pdf]

Patterns, Volume *4*

Supplemental information

Evaluating vaccine allocation strategies using simulation-assisted causal modeling

Armin Kekić, Jonas Dehning, Luigi Gresele, Julius von Kügelgen, Viola Priesemann, and Bernhard Schölkopf

Supplemental Information

A. Supplemental Experimental Procedures

A.1. Generating vaccine allocation strategies

A.1.1. Factual strategy

In the available dataset [1] the vaccination times for first, second and third doses are given for each age group, *i.e.*

$$P(T_i|A) \quad \text{for } i = 1, 2, 3. \tag{43}$$

However, this alone does not uniquely determine the distribution of waning times. To fully specify required joint distributions $P(T_1, T_2|A)$ and $P(T_3|T_2, A)$ we employ a greedy algorithm to

1. Maximise the number of vaccine recipients that receive their second dose 3 weeks after their initial dose (or as close to 3 weeks as possible, but not sooner).
2. Have a minimum gap of 12 weeks between second and third dose (constraint).
3. Satisfy (43) (constraint).

A.1.2. Uniform strategy

For all counterfactual strategies we require the number of first, second and third doses to match the factual—but not in each age group. In other words,

$$\tilde{P}(T_i) = P(T_i) \quad \text{for } i = 1, 2, 3. \tag{44}$$

where the tilde indicates probabilities in the counterfactual scenario. For the `Uniform` strategy, we require all age groups to have the same vaccination time distributions:

$$\tilde{P}(T_1, T_2|A) = \tilde{P}(T_1, T_2) \quad \text{and} \quad \tilde{P}(T_3|T_2, A) = \tilde{P}(T_3|T_2). \tag{45}$$

In summary, the greedy algorithm to determine $\tilde{P}(T_1, T_2|A)$ and $\tilde{P}(T_3|T_2, A)$ is adapted as follows:

1. Maximise the number of vaccine recipients that receive their second dose 3 weeks after their initial dose (or as close to 3 weeks as possible, but not sooner).
2. Have a minimum gap of 12 weeks between second and third dose (constraint).
3. Satisfy (44) (constraint).
4. Satisfy (45) (constraint).

A.1.3. Ranked strategies

In the ranked strategies `ElderlyFirst`, `YoungFirst`, `RiskRanked` and `RiskRankedReversed` all age groups are ranked and vaccinations are assigned to the highest-ranked age group its vaccine uptake rate is reached. The counterfactual vaccine uptake rate per age group is the factual one for the first and second doses, *i.e.*

$$\sum_{t_i=1}^{M} \tilde{P}(t_i|a) \leq \sum_{t_i=1}^{M} P(t_i|a) \quad \text{for } i = 1, 2 \text{ and } \forall a. \tag{46}$$

The vaccine uptake rate for booster shots is slightly relaxed by 2.5% to meet other consistency constraints:

$$\sum_{t_3=1}^{M} \tilde{P}(t_3|a) \leq 0.025 + \sum_{t_3=1}^{M} P(t_3|a) \quad \forall a. \tag{47}$$

The greedy algorithm is adapted to

1. Prioritise age groups according to ranking.
2. Maximise the number of vaccine recipients that receive their second dose 3 weeks after their initial dose (or as close to 3 weeks as possible, but not sooner).
3. Have a minimum gap of 12 weeks between second and third dose (constraint).
4. Satisfy (44) (constraint).
5. Satisfy (46) and (47) (constraint).

A.2. Target function

Our goal is to compute the expected number of severe cases after intervening on the distribution of vaccination times:

$$s(\tilde{\pi} = \tilde{P}(T_1, T_2, T_3|A)) := MD\, \mathbb{E}[S|\text{do}(T_1, T_2, T_3 \sim \tilde{P}(T_1, T_2, T_3|A))] \tag{48}$$

where $\tilde{P}(T_1, T_2, T_3|A)$ is the distribution of vaccination times in the counterfactual scenario. Using results for stochastic policies from [2], repeated application of the law of total probabilities (TP) and exploiting conditional independences (CI) implied by the causal graph (see Figure 2) we can derive an expression for (48) in terms of known conditional probabilities:

$$\begin{aligned} & s(\tilde{\pi} = \tilde{P}(T_1, T_2, T_3|A)) \\ & := MD\ \mathbb{E}[S|\text{do}(T_1, T_2, T_3 \sim \tilde{P}(T_1, T_2, T_3|A))] && (49) \\ & = MD\ P\left(S{=}1|\text{do}(T_1, T_2, T_3 \sim \tilde{P}(T_1, T_2, T_3|A))\right) && (50) \end{aligned}$$

$$\stackrel{[2, \text{ Ch. } 4.2]}{=} MD \sum_a P(a) \sum_{t_1,t_2,t_3=1}^{M+1} \tilde{P}(t_1,t_2,t_3|a)$$

$$P(S{=}1|\mathrm{do}(t_1,t_2,t_3),a) \tag{51}$$

$$\stackrel{\mathrm{TP}}{=} MD \sum_a P(a) \sum_{t_1,t_2,t_3=1}^{M+1} \tilde{P}(t_1,t_2,t_3|a) \sum_{t=1}^{M} \underbrace{P(t|\mathrm{do}(t_1,t_2,t_3),a)}_{\stackrel{\mathrm{CI}}{=} P(t)}$$

$$P(S{=}1|\mathrm{do}(t_1,t_2,t_3),a,t) \tag{52}$$

$$\stackrel{\mathrm{TP}}{=} MD \sum_a P(a) \sum_{t=1}^{M} \underbrace{P(t)}_{=1/M} \sum_{t_1,t_2,t_3=1}^{M+1} \tilde{P}(t_1,t_2,t_3|a)$$

$$\sum_{w=1}^{M} \underbrace{P(w|\mathrm{do}(t_1,t_2,t_3),a,t)}_{\stackrel{\mathrm{CI}}{=} P(w|\mathrm{do}(t_1,t_2,t_3),t)} P(S{=}1|\mathrm{do}(t_1,t_2,t_3),a,t,w) \tag{53}$$

$$\stackrel{\mathrm{TP}}{=} D \sum_a P(a) \sum_{t=1}^{M} \sum_{t_1,t_2,t_3=1}^{M+1} \tilde{P}(t_1,t_2,t_3|a) \sum_{w=1}^{M} P(w|\mathrm{do}(t_1,t_2,t_3),t)$$

$$\sum_{v=0}^{3} \underbrace{P(v|\mathrm{do}(t_1,t_2,t_3),a,t,w)}_{\stackrel{\mathrm{CI}}{=} P(v|\mathrm{do}(t_1,t_2,t_3),t)} \underbrace{P(S{=}1|\mathrm{do}(t_1,t_2,t_3),v,a,t,w)}_{\stackrel{\mathrm{CI}}{=} P(S=1|v,a,t,w)} \tag{54}$$

$$= D \sum_a P(a) \sum_{t=1}^{M} \sum_{t_1,t_2,t_3=1}^{M+1} \tilde{P}(t_1,t_2,t_3|a) \sum_{w=1}^{M} P(w|\mathrm{do}(t_1,t_2,t_3),t)$$

$$\sum_{v=0}^{3} P(v|\mathrm{do}(t_1,t_2,t_3),t)\ P(S{=}1|v,a,t,w) \tag{55}$$

$$= D \sum_a P(a) \sum_{t=1}^{M} \sum_{t_1,t_2,t_3=1}^{M+1} \tilde{P}(t_1,t_2,t_3|a) \sum_{w=1}^{M} P(w|t_1,t_2,t_3,t)$$

$$\sum_{v=0}^{3} P(v|t_1,t_2,t_3,t)\ P(S{=}1|v,a,t,w). \tag{56}$$

In the last step we use that since $\{T_1, T_2, T_3, T\}$ are the parents of V and W we can replace the do-operators by conditional probabilities [2]. Also note that for notational convenience, we set the vaccination time $t_i = M + 1$ for a patient who has not received the ith dose during the considered time window $t \in \{1, \ldots, M\}$.

The waning time w (number of weeks since the last dose was received) depends deter-

ministically on the vaccination times t_1, t_2, t_3:

$$P(w|t_1,t_2,t_3,t) = \begin{cases} 1 & \text{if } w = \max_{i\in\{1,2,3\}}[t-t_i]^+, \\ 0 & \text{else,} \end{cases}$$
$$\text{where } [x]^+ = \begin{cases} x & \text{if } x \geq 0, \\ 0 & \text{else.} \end{cases} \tag{57}$$

The vaccination status v also depends deterministically on the vaccination times t_1, t_2, t_3:

$$P(v|t_1,t_2,t_3,t) = \begin{cases} 1 & \text{if } v = \max_{i\in\{0,1,2,3\}} \left(i \cdot \operatorname{sgn}(t-t_i+1)\right), \\ 0 & \text{else,} \end{cases} \tag{58}$$

where sgn is the sign function. These deterministic relationships can be used to eliminate the corresponding conditionals from the target function:

$$\begin{aligned}
&s(\tilde{\pi} = \tilde{P}(T_1,T_2,T_3|A)) \\
&= D \sum_a P(a) \sum_{t=1}^{M} \\
&\times \Bigg[\sum_{t_1=t+1}^{M+1} \sum_{t_2=t+1}^{M+1} \sum_{t_3=t+1}^{M+1} \tilde{P}(t_1,t_2,t_3|a)\ P(S{=}1|t,a,v{=}0,w{=}0) \\
&+ \sum_{t_1=1}^{t} \sum_{t_2=t+1}^{M+1} \sum_{t_3=t+1}^{M+1} \tilde{P}(t_1,t_2,t_3|a)\ P(S{=}1|t,a,v{=}1,w{=}(t-t_1)) \\
&+ \sum_{t_1=1}^{t} \sum_{t_2=1}^{t} \sum_{t_3=t+1}^{M+1} \tilde{P}(t_1,t_2,t_3|a)\ P(S{=}1|t,a,v{=}2,w{=}(t-t_2)) \\
&+ \sum_{t_1=1}^{t} \sum_{t_2=1}^{t} \sum_{t_3=1}^{t} \tilde{P}(t_1,t_2,t_3|a)\ P(S{=}1|t,a,v{=}3,w{=}(t-t_3)) \Bigg] .
\end{aligned} \tag{59}$$

Substituting the factorisation for the severity mechanism $P(S{=}1|v,a,t,w)$ we get:

$$
\begin{aligned}
&s(\tilde{\pi} = \tilde{P}(T_1, T_2, T_3|A)) \\
&= D \sum_a P(a) \sum_{t=1}^{M} f^0(t) f^1_{\tilde{\pi}}(a,t) \\
&\quad \times \Bigg[\sum_{t_1=t+1}^{M+1} \sum_{t_2=t+1}^{M+1} \sum_{t_3=t+1}^{M+1} \tilde{P}(t_1,t_2,t_3|a)\ g(0,a) \\
&\quad + \sum_{t_1=1}^{t} \sum_{t_2=t+1}^{M+1} \sum_{t_3=t+1}^{M+1} \tilde{P}(t_1,t_2,t_3|a)\ g(1,a)\, h^1(t-t_1) \\
&\quad + \sum_{t_1=1}^{t} \sum_{t_2=1}^{t} \sum_{t_3=t+1}^{M+1} \tilde{P}(t_1,t_2,t_3|a)\ g(2,a)\, h^2(t-t_2) \\
&\quad + \sum_{t_1=1}^{t} \sum_{t_2=1}^{t} \sum_{t_3=1}^{t} \tilde{P}(t_1,t_2,t_3|a)\ g(3,a)\, h^3(t-t_3) \Bigg] .
\end{aligned} \tag{60}
$$

B. Base reproduction number estimates

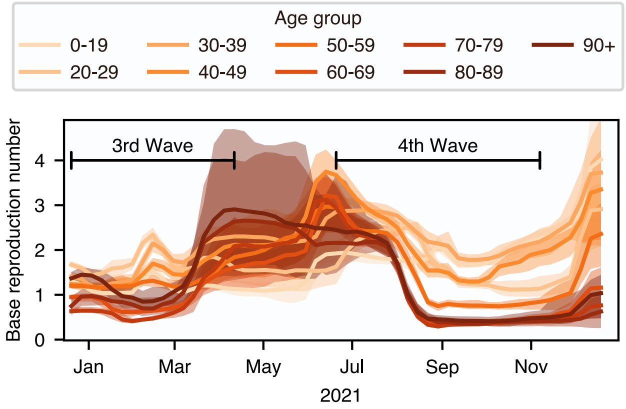

Figure S1: **Base reproduction numbers per age group inferred by the SEIR-like infection dynamics model.** The base reproduction numbers describe the contribution of each age group to infection spread after accounting for the effect of vaccination. The plot shows inferred weekly base reproduction numbers between 2020-12-20 and 2021-12-25. The shaded areas show the 95% credible intervals. The periods with high uncertainty coincide with low-incidence periods.

C. Results assuming other mixing factors

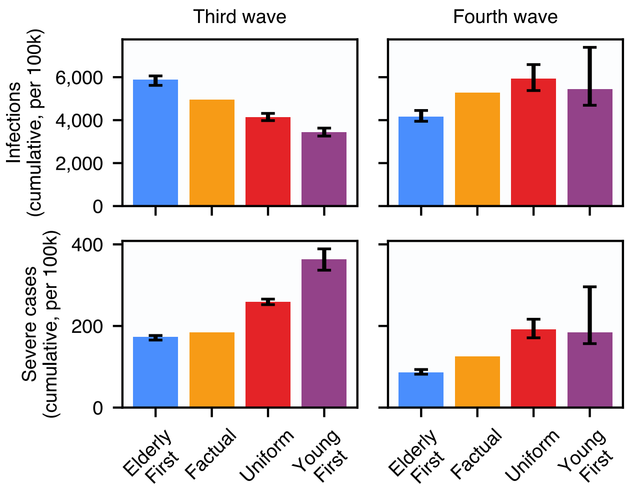

Figure S2: **Cumulative incidences of infections (top row) and severe cases (bottom row) for the two infection waves in 2021 under the factual and counterfactual vaccine allocation strategies, assuming a contact mixing factor of 0.7.** For the third wave we sum all cases from 2020-12-20 to 2021-04-11; for the fourth wave from 2021-06-20 to 2021-11-07. The whiskers show the 95% credible intervals.

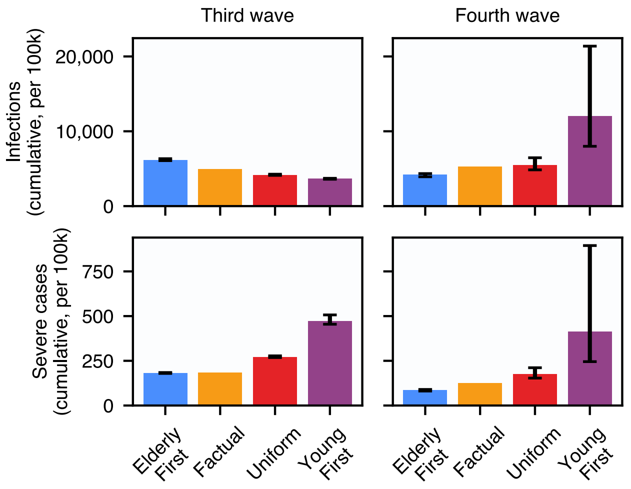

Figure S3: **Cumulative incidences of infections (top row) and severe cases (bottom row) for the two infection waves in 2021 under the factual and counterfactual vaccine allocation strategies, assuming a contact mixing factor of** 0.9**.** For the third wave we sum all cases from 2020-12-20 to 2021-04-11; for the fourth wave from 2021-06-20 to 2021-11-07. The whiskers show the 95% credible intervals.

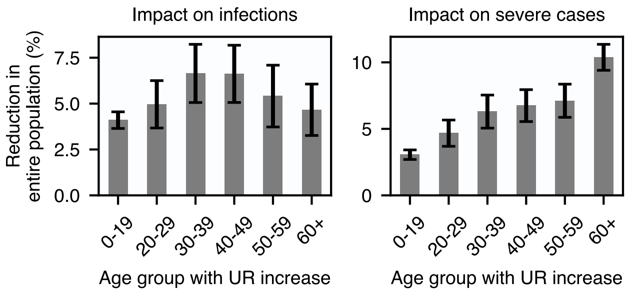

Figure S4: **Impact of increasing vaccine uptake rate (UR) in a given age group on severe cases, assuming a contact mixing factor of** 0.7**.** In each scenario, the vaccine uptake rate is increased in a given age group by a fixed number corresponding to 0.6% of the population being motivated to get vaccinated. We assume that the change comes from originally unvaccinated individuals who are persuaded to receive three doses. The plots show the impact on cumulative infections (left) and severe cases (right) in the entire population—not just in the age group in which the UR was increased. We consider cases from 2020-12-20 to 2021-12-25. The whiskers show the 95% credible intervals.

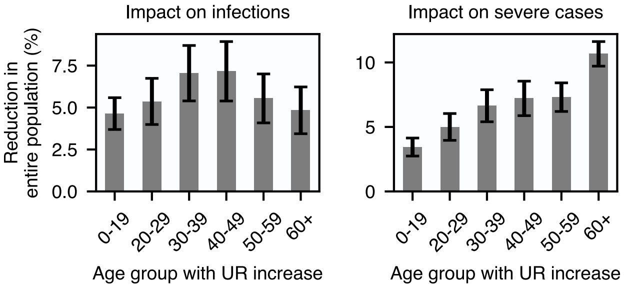

Figure S5: **Impact of increasing vaccine uptake rate (UR) in a given age group on severe cases, assuming a contact mixing factor of** 0.9**.** In each scenario, the vaccine uptake rate is increased in a given age group by a fixed number corresponding to 0.6% of the population being motivated to get vaccinated. We assume that the change comes from originally unvaccinated individuals who are persuaded to receive three doses. The plots show the impact on cumulative infections (left) and severe cases (right) in the entire population—not just in the age group in which the UR was increased. We consider cases from 2020-12-20 to 2021-12-25. The whiskers show the 95% credible intervals.

References

[1] Ministry of Health Israel. *COVID-19 Database*.

[2] Judea Pearl. *Causality*. Cambridge University Press, 2009. DOI: https://doi.org/10.1017/CBO9780511803161
