# Supplementary material for: Evaluating vaccine allocation strategies using simulation-assisted causal modeling
Source: Patterns (N Y). 2023 May 3;4(6):100739. doi: 10.1016/j.patter.2023.100739 (PMC10155501; doi:10.1016/j.patter.2023.100739)
Supplement: Document S2. Article plus supplemental information [file mmc2.pdf]

Article

Patterns

Evaluating vaccine allocation strategies using simulation-assisted causal modeling

Highlights

- Evaluated vaccination strategies using causality and infection dynamics simulations
- Compared Israel's implemented strategy for COVID-19 with counterfactual scenarios
- Different age groups need prioritization based on target (infections/severe cases)
- The modular structure enables easy adaptation of the model to future pandemics

Authors

Armin Kekić, Jonas Dehning, Luigi Gresele, Julius von Kügelgen, Viola Priesemann, Bernhard Schölkopf

Correspondence

armin.kekic@tuebingen.mpg.de

In brief

Kekić et al. combine causal modeling, infection dynamics simulations, and literature estimates of immunity waning to evaluate vaccine allocation strategies during pandemics. Their study of COVID-19 shows that prioritizing middle-aged groups reduces infections, while prioritizing elderly groups minimizes severe cases most effectively. This work also highlights the impact of waning immunity and timing of vaccination campaigns on vaccine allocation effectiveness, providing insights for pandemic response.

Kekić et al., 2023, Patterns *4*, 100739
June 9, 2023 © 2023 The Author(s).
https://doi.org/10.1016/j.patter.2023.100739

CellPress

CellPress
OPEN ACCESS

Article

Evaluating vaccine allocation strategies using simulation-assisted causal modeling

Armin Kekić,[1,6,*] Jonas Dehning,[2] Luigi Gresele,[1] Julius von Kügelgen,[1,3] Viola Priesemann,[2,4,5] and Bernhard Schölkopf[1,5]
[1]Empirical Inference Department, Max Planck Institute for Intelligent Systems, 72076 Tübingen, Germany
[2]Max Planck Institute for Dynamics and Self-Organization, 37077 Göttingen, Germany
[3]Department of Engineering, University of Cambridge, Cambridge CB2 1PZ, UK
[4]Department of Physics, Georg August University, 37077 Göttingen, Germany
[5]Senior author
[6]Lead contact
*Correspondence: armin.kekic@tuebingen.mpg.de
https://doi.org/10.1016/j.patter.2023.100739

THE BIGGER PICTURE Learning about a complex system and simulating alternative scenarios under changed conditions or dynamics is a challenging problem. Consider the time evolution of COVID-19 cases, which depends on a combination of contact patterns, demographics, and vaccination rates. How many severe cases could have been prevented had a different vaccine allocation strategy been implemented? To answer such counterfactual questions, we propose an approach that merges (1) coarse-grained causal modeling, (2) ordinary-differential-equation-based simulation, and (3) domain knowledge, combining the advantages of different modeling paradigms. The resulting hybrid model can be viewed as a "causal digital twin" of the underlying complex system; it captures relevant features thereof and allows reasoning about novel scenarios and interventions. We hope that our hybrid causal approach can inspire modeling for other domains where causal reasoning about a complex system is of interest.

1 2 3 4 5 **Proof-of-Concept:** Data science output has been formulated, implemented, and tested for one domain/problem

SUMMARY

We develop a model to retrospectively evaluate age-dependent counterfactual vaccine allocation strategies against the coronavirus disease 2019 (COVID-19) pandemic. To estimate the effect of allocation on the expected severe-case incidence, we employ a simulation-assisted causal modeling approach that combines a compartmental infection-dynamics simulation, a coarse-grained causal model, and literature estimates for immunity waning. We compare Israel's strategy, implemented in 2021, with counterfactual strategies such as no prioritization, prioritization of younger age groups, or a strict risk-ranked approach; we find that Israel's implemented strategy was indeed highly effective. We also study the impact of increasing vaccine uptake for given age groups. Because of its modular structure, our model can easily be adapted to study future pandemics. We demonstrate this by simulating a pandemic with characteristics of the Spanish flu. Our approach helps evaluate vaccination strategies under the complex interplay of core epidemic factors, including age-dependent risk profiles, immunity waning, vaccine availability, and spreading rates.

INTRODUCTION

The coronavirus disease 2019 (COVID-19) pandemic posed significant challenges to societies and decision makers around the world. Many governments implemented non-pharmaceutical interventions to limit the spread of infections and reduce the number of severe cases.[1,2] The development of efficient vaccines has provided another key control measure to combat the COVID-19 pandemic.[3] However, vaccine supply can fail to meet demand, and vaccine uptake can fall short of expectations. Under these conditions, governments have to find rational strategies to allocate vaccines to minimize harm. It is therefore important to understand how to evaluate and compare different vaccine allocation strategies.[4]

One crucial aspect to consider when designing such strategies is age, which is a key risk factor for COVID-19 mortality.[5]

This is an open access article under the CC BY license (http://creativecommons.org/licenses/by/4.0/).

While pre-existing conditions and the high exposure of health care workers have also played a role in vaccine prioritization,[6,7] in this work, we focus on age dependence, including interactions between age groups, as one of the most important factors. Given an observed evolution of infections and severe cases, we seek to answer central policy questions. Given limited vaccine availability, should one have prioritized first vaccinations of the young or booster shots for the elderly? Which age groups should have been targeted preferentially to increase vaccine uptake? How would a different age-dependent risk profile have impacted the outcome of a vaccination policy? Answering such questions requires computing the effects of hypothetical interventions on a complex system (given observations of the same system under different conditions). Such "what if" scenarios lie at the heart of causal inference and relate to counterfactual reasoning; that is, reasoning about how events would have turned out had some circumstances been altered.[8,9]

The gold standard for inferring average causal effects are randomized controlled trials (RCTs),[10] which are used to evaluate medical treatments such as COVID-19 vaccines.[11] However, to compare full vaccine allocation strategies at the country level, running an RCT is infeasible in practice (there is only one copy of each country), mirroring similar challenges in the empirical estimation of individualized treatment effects.[12,13] Moreover, it would be ethically unacceptable to implement vaccine allocation strategies that are expected to be suboptimal for the population. Here, modeling approaches provide an important tool to fill this gap.[14] For COVID-19, one and a half years into the vaccination campaign, we now have data (age-resolved cases, hospitalizations, and vaccination times) to infer the reduction of spread because of vaccination across different age groups and subsequently simulate counterfactual vaccination scenarios.

To capture the effects of changes in vaccine allocation strategy, we have to model their impact on spread and hospitalization. Furthermore, we need to consider aspects such as vaccine efficacy, immunity waning, age-dependent risk profiles, and contact structures. Two established modeling paradigms are compartmental differential equation models and machine learning (ML) approaches.

In principle, compartmental models like the susceptible-infectious-recovered (SIR) model (and its extensions) can be used to answer the types of questions in which we are interested, provided that all relevant parameters are known sufficiently well.[15–19] However, this is typically not the case. Thus, a framework combining inference of parameters and prediction is necessary. To jointly model infections and severe cases, compartmental models require a large state space whose parameters can be difficult to estimate from data without overfitting.[20]

ML methods excel at fitting data and making predictions based on statistical associations but are generally unable to answer causal questions. Moreover, they are unreliable when the underlying data distribution changes. Yet, we are precisely interested in how our system behaves under distribution shifts; we want to know how the expected severe-case incidence would have changed had we implemented different vaccine allocation strategies.

Causal models occupy a middle ground between the two paradigms and are better suited for our purpose because they are modular and interventional. Modularity refers to a model being composed of autonomous components or subsystems and allows combining different sources of knowledge. Interventions are naturally supported by causal models because they explicitly capture the data-generating mechanisms rather than mere statistical associations. Causal models can thus answer counterfactual questions while clearly expressing the underlying assumptions.[8,21] However, using them to describe time-varying systems and modeling the dynamics of epidemic spread is cumbersome.

Because neither compartmental nor causal models on their own are fully suited for our task, we resort to a hybrid modeling approach involving a modular combination thereof. We propose a coarse-grained causal model in which most components are estimated from data but where, additionally, one of the modules is a compartmental model and another one is derived from literature estimates. In particular, we use a susceptible-exposed-infectious-recovered (SEIR) model for the infection dynamics, but not for severe cases, and rely on literature estimates of immunity waning, as illustrated in Figure 1. The SEIR-like infection dynamics model can be fit more easily to data compared with a joint model of severe cases and infections. For severe cases, given a set of qualitative causal assumptions, fitting the causal model reduces to the problem of statistical estimation of conditional probabilities. This combines the strengths of the two approaches: the data-driven nature of causal models and the expressivity of compartmental models.

As a case study, we apply our method to a comprehensive dataset collected in Israel.[22] Specifically, we compare several counterfactual age-dependent vaccine allocation strategies with the factual strategy, assuming a fixed number of administered doses and fixed vaccine uptake rate per age group. We also simulate the effect of campaigning for vaccine uptake in a given age group by increasing the vaccine uptake rate in one group and computing the effect on the severe-case incidence across all age groups. To showcase the capability of our model to change and examine the influence of individual modules, we consider a different type of disease whose age-dependent risk profile is based on the Spanish flu. We also investigate the effect of waning immunity by changing the timescale at which immunity weakens.

RESULTS

Methods summary

We use a causal graphical model,[8] as shown in Figure 2, to describe an individual's probability of developing severe COVID-19. We use a binary variable, $S \in \{0,1\}$, where 1 indicates a severe case and 0 describes a mild case or no infection at all. We assume that the severe-case probability depends on the following variables: the vaccination status of the individual, $V \in \{0,1,2,3\}$, indicating the number of vaccine doses a person has received; their age group, $A \in \{0-19, 20-29, \ldots, 80-89, 90+\}$; the current week, $T \in \{1, \ldots, M\}$; and the waning time (i.e., the time since the last dose was received), $W \in \{1, \ldots, M\}$. M is the number of weeks in the considered time window. The variables V and W are functions of the weeks in which the respective doses were received, $T_i \in \{1, \ldots, M+1\}$ for $i \in \{1,2,3\}$ and the current week T. By common convention,

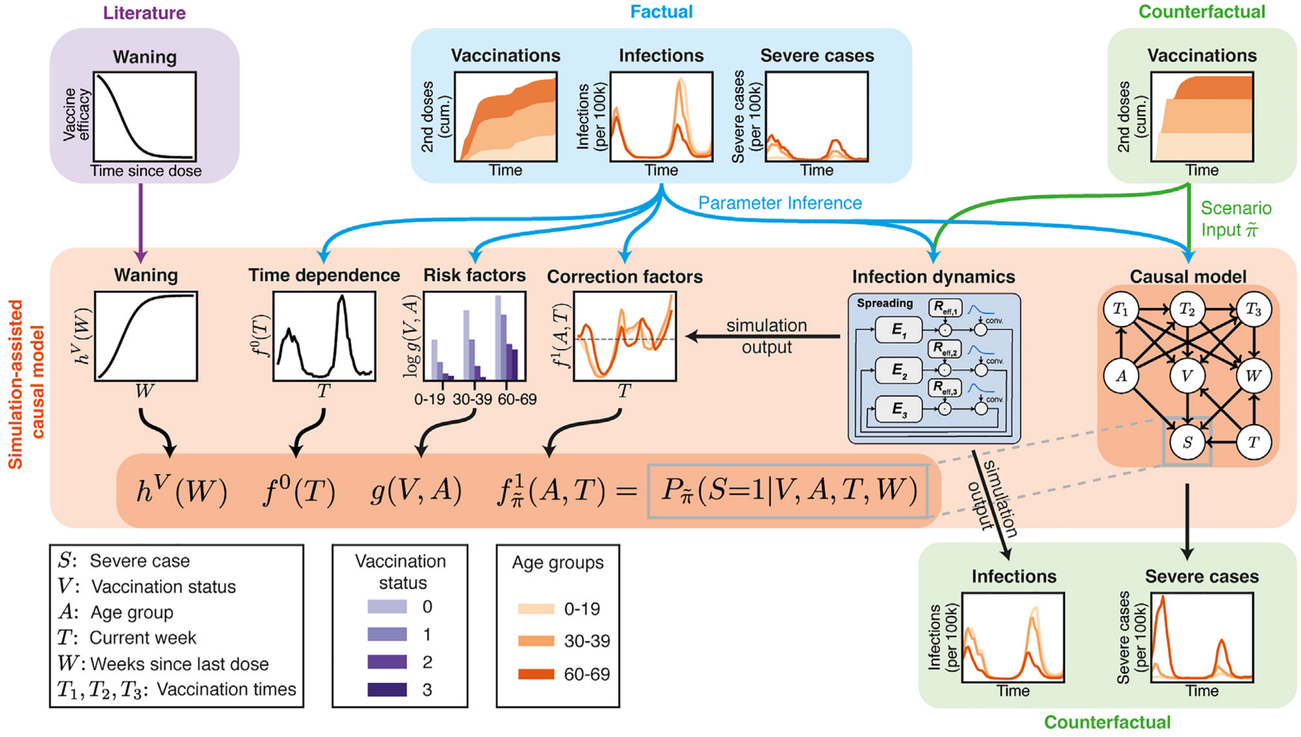

Figure 1. Method overview

Our goal is to compute the weekly severe-case incidence (bottom right) under counterfactual vaccine allocation strategies (in the example shown here: `YoungFirst`, top right). To compute this counterfactual scenario, we provide an estimate of the severity mechanism $P_{\tilde{\pi}}(S = 1|V,A,T,W)$ for all combinations of (V,A,T,W) through our proposed factorization (2). The other conditionals in the causal graph are directly estimated from data (factual strategy, top center) or are intervened upon according to the counterfactual strategy. The risk factors $g(V,A)$ and the time dependence $f^0(T)$ are estimated from data after accounting for immunity waning $h(W)$ derived from literature estimates (top left). An SEIR-like infection dynamics model is fit to the factual infections and subsequently used to simulate infections under the counterfactual strategy. The simulation output is used to compute the correction factors $f^1_{\tilde{\pi}}(A,T)$ accounting for the age-specific change in probability of being infected. Only three of nine age groups are shown for simplicity.

we denote random variables by uppercase letters and realizations thereof by lowercase letters.

We are interested in how interventions on the distribution of vaccination times for different age groups A affect the expected severe-case probability:

$$\mathbb{E}[S|\mathrm{do}(T_1,T_2,T_3 \sim \tilde{P}(T_1,T_2,T_3|A))] \quad \text{(Equation 1)}$$

where we denote the distributions of vaccination times pre and post intervention as $\pi = P(T_1,T_2,T_3|A)$ and $\tilde{\pi} = \tilde{P}(T_1, T_2, T_3|A)$, respectively. The $\mathrm{do}(\cdot)$ operator describes a change in distribution arising from an intervention[8] (see Target function for details).

There are three challenges we have to address. (1) In the publicly available data by Israel's Ministry of Health,[22] severe outcomes are not registered as a function of time since the last dose was received. We only have access to the marginal distribution $P(S|V, A, T) = \sum_w P(S|V, A, T, w)P(w|V, A, T)$. (2) Computing the post-intervention severity (1) involves evaluating the conditional $P(S|V,A,T)$ for combinations (V,A,T), for which there are no observations. In particular, because of the implemented age-ranked vaccine allocation strategy in Israel, there may not have been any vaccinated subjects in certain younger age groups for some of the early weeks. (3) Changing the vaccine allocation strategy influences the probability of having a severe case in two ways: first, by changing the probability of having immunity through vaccination, and second, through impacting the infection dynamics at the population level. Such changes in infection dynamics are not captured by the causal model alone.

To address these challenges, we propose a factorization of the severity mechanism

$$P_{\tilde{\pi}}(S = 1|V,A,T,W) = f^0(T)\, g(V,A)\, h^V(W)\, f_{\tilde{\pi}}^{\,1}(A,T). \quad \text{(Equation 2)}$$

The observed aggregate time dependence of the probability of having a severe case is captured by $f^0(T)$. The factor $g(V,A)$ describes the age- and vaccination-status-dependent relative risk factor of having a severe case, where we normalize $g = 1$ for the unvaccinated 60- to 69-year-olds. The factor $h^V(W)$ describes the waning of immunity against infection. Finally, $f_{\tilde{\pi}}^{\,1}(A,T)$ is a correction factor that depends on the post-intervention vaccination distribution and accounts for the change in infection dynamics. The subscript $\tilde{\pi}$ indicates factors that depend on the post-intervention vaccine allocation strategy.

The factorization (2) resolves the challenges above by allowing us to (1) incorporate literature knowledge about immunity waning into our causal model; (2) estimate $P(S = 1|V,A,T,W)$ for all values in the conditioning set by transferring knowledge between

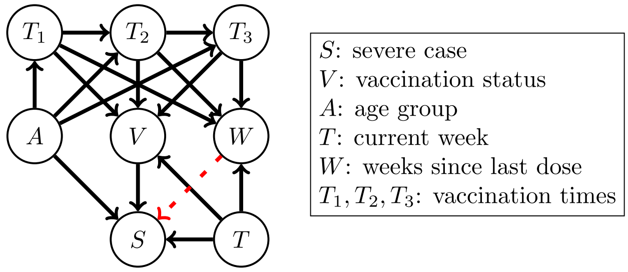

Figure 2. Causal graph used to model the variables influencing severe COVID-19 cases S

We consider the variables vaccination status V, age group A, the current week T, and the time since the last dose was administered W. V and W deterministically depend on the current week T and the vaccination times T_1, T_2, T_3 for each dose. An arrow indicates that one variable has a direct causal influence on another. Note that the age group A influences V and W indirectly via the vaccination times T_1, T_2, T_3. The red dashed arrow (red dashed arrow) indicates a relationship that cannot be estimated because the data are incomplete.

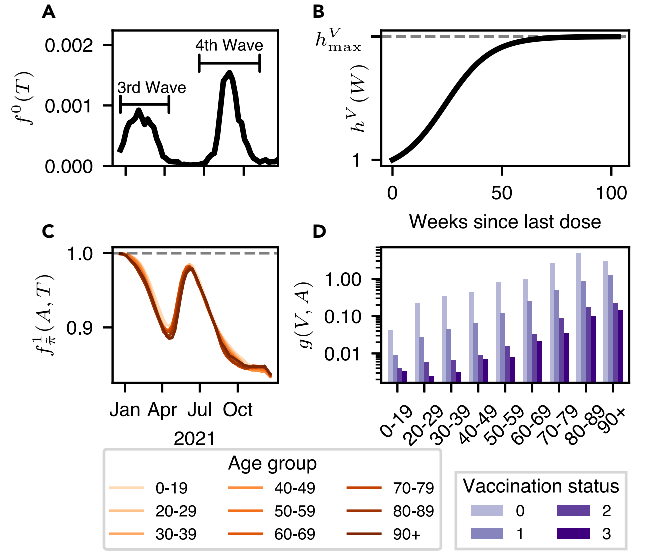

Figure 3. Factors contributing to the severe case probability

(A) Estimated time dependence $f^0(T)$ approximately following the two infection waves in Israel in 2021.

(B) The waning curve $h^V(W)$ with increasing risk over time as immunity wanes. The waning curve is computed based on results from Tartof et al.[23]

(C) Estimated age-dependent correction factors $f^1_{\tilde{\pi}}(A,T)$ accounting for the change in population-level infection dynamics. The correction factors shown here correspond to the scenario with increased vaccine uptake rate by 2%, leading to a relative decrease in the number of infections.

(D) Risk factor $g(V,A)$ estimates indicating the relative risk of having a severe case by age A and vaccination status V.

vaccination states, age groups, and weeks; and (3) take into account the population-level impact on the infection dynamics.

$h^V(W)$ can be derived from literature estimates for the vaccine efficacy against infection as a function of time since the last dose was administered.[23] $f^0(T)$ and $g(V,A)$ can be estimated from data after correcting for the influence of waning. The correction factor $f_{\tilde{\pi}}^{\,1}(A,T)$ under the counterfactual vaccine allocation strategy is given by the relative change in weekly infection probability for each age group

$$f_{\tilde{\pi}}^{\,1}(A,T) = \frac{P_{\tilde{\pi}}(I=1|V,A,T,W)}{P_{\pi}(I=1|V,A,T,W)}. \qquad \text{(Equation 3)}$$

Hence, the correction factor couples the compartmental model for infection dynamics with the causal model. The derivations of all estimators are given in estimating the severity mechanism factors. Estimated factors are shown in Figure 3.

To estimate the effect of changing the vaccine allocation strategy on the infection dynamics ($P_{\tilde{\pi}}(I = 1|V,A,T,W)$), we first infer the parameters of a Bayesian SEIR-like model under the factual strategy π. Vaccines are assumed to offer some protection against infection: 70%, 90%, and 95% directly after the first, second, and third dose, respectively, after which the protection is waning at the same rate.[23] For each age group, we fit a time-dependent base reproduction number; that is, the reproduction number in a hypothetical non-immune population (Figure 4 left). We assume a generation interval of 4 days[24,25] and a reporting delay of 6 days. Non-equal reproduction numbers for every age group are modeled by modulating symmetrically the rows and columns of a contact matrix. The preference for contacts within each age group is parameterized by a contact mixing factor γ between 0 (no mixing between age groups) and 1 (all-to-all connections). By default, this factor is set to 0.8; we show that results are similar with lower and higher mixing factors in supplement C. Because our method estimates overall contact strengths per age group through the reproduction numbers, diary-based estimates of the contact patterns are not appropriate in this setting (for details, see The contact matrix). With the inferred reproduction number, we rerun the model with the counterfactual vaccine allocation strategy $\tilde{\pi}$ to obtain an estimate of the number of infections in the counterfactual scenario. The simulated number of infections is then used to calculate the correction factor (3).

Compartmentalizing the factors related to severe cases into the causal model simplifies parameter estimation; for the causal model, we need to estimate conditional probabilities, which is trivial given our data (except for the severity mechanism [2]). This reduces the state space and the parameters that need to be estimated for the compartmental infection dynamics model.

Counterfactual vaccine allocation strategies

We compare four age-dependent vaccine allocation strategies: the Factual and three counterfactual ones. (1) `Factual`: the vaccine allocation strategy implemented in Israel generally prioritized the elderly (starting with all people aged 60 and over) for the initial two doses and for booster shots but also prioritized nursing home residents, patients with pre-existing medical conditions, and frontline health care workers.[6] (2) `Uniform`: in the uniform strategy, we do not implement any prioritization based on age or any other factors; all age groups are vaccinated at the same rate. (3) `ElderlyFirst`: we prioritize age groups in descending order, starting with the oldest and ending with the youngest. This strategy differs from the Factual strategy in that it strictly prioritizes by age and does not consider other factors. (4) `YoungFirst`: the opposite of `ElderlyFirst`. The counterfactual strategies are simulated, while, here, the outcome of the `Factual` strategy is taken

CellPress
OPEN ACCESS

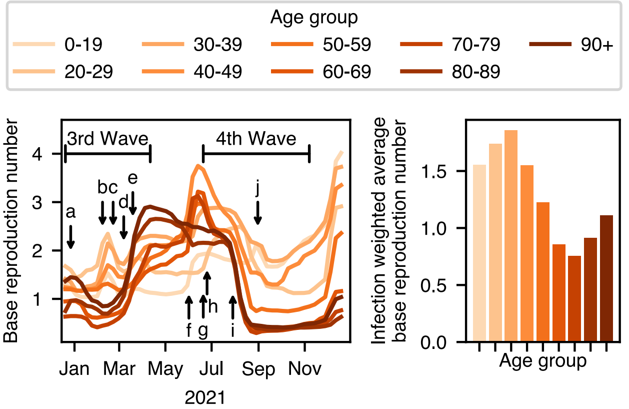

Figure 4. Base reproduction numbers per age group inferred by the SEIR-like infection dynamics model

The base reproduction numbers describe the contribution of each age group to infection spread after accounting for the effect of vaccination. Left: inferred weekly base reproduction numbers between December 20, 2020 and December 25, 2021. a, lockdown;[26] b–e, restriction-easing phases 1–4;[27] f, Green pass and purple badge requirement lifted; g, high and middle school summer break start; h, indoor mask requirements;[28] i, green pass requirements;[29] j, school summer break end. The ticks on the x axis indicate the first day of the respective month. Right: average base reproduction numbers weighted by the factual total weekly infections. Note that uncertainty on the base reproduction number is high during spring 2021 because of low case numbers (Figure S1). The 90+ age group has only 3–4 cases per week on average during this period.

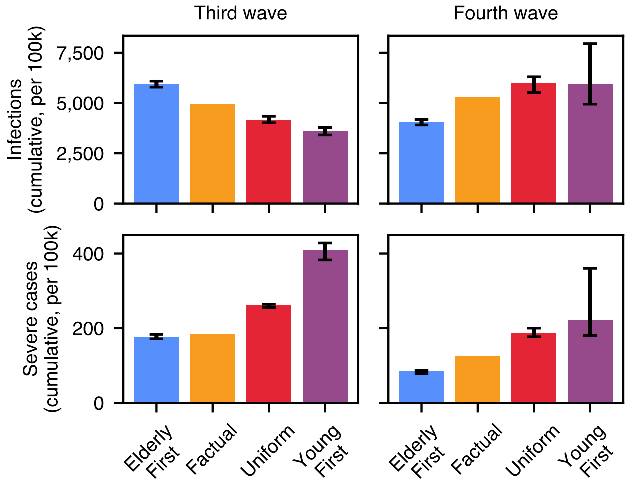

Figure 5. Cumulative incidence of infection and severe cases for the two infection waves in 2021 under the factual and counterfactual vaccine allocation strategies

For the third wave, we sum all cases from December 20, 2020 to April 11, 2021 and for the fourth wave from June 20, 2021 to November 7, 2021. The whiskers show the 95% credible intervals. The outcome of the `Factual` strategy is taken from data,[22] while the other vaccine allocation strategies are simulated.

from data. In the simulated scenarios, the number of first, second, and third doses is kept fixed to the factual numbers. The vaccine uptake rate per age group is also fixed to the factual values up to a margin of error of 2.5 percentage points to be able to satisfy other constraints, such as the total number of vaccines or minimum times between doses (see supplement A.1 for more details).

Infections

As shown in Figure 5, during the third wave, `YoungFirst` leads to the lowest infection incidence, followed by the `Uniform` and the `Factual` strategy. `ElderlyFirst` leads to the highest infection incidence.

The most effective strategy in preventing infections is determined by which age group is contributing most to infection spread. The base reproduction numbers express how much an age group contributes to infection spread after removing the effect of vaccinations; in other words, how much an age group would contribute to infection spread if no one in that age group were vaccinated. As shown in Figure 4, during the third wave, the base reproduction numbers tended to be higher among the young and middle-aged groups and lower for the elderly, pointing to differences in behavior and average number of contacts. Therefore, strategies that prioritize young and middle-aged groups are most effective at preventing infections.

During the fourth wave, the relative effectiveness of strategies at preventing infections is reversed. `ElderlyFirst` leads to the lowest infection incidence, followed by the `Factual` strategy. `YoungFirst` and `Uniform` lead to the most number of infections.

The order of the vaccine allocation strategies regarding infections in the fourth wave is changed because the estimated base reproduction numbers per age group are different. For the first half of the wave, the base reproduction number in the youngest age group, accounting for roughly a third of the population, tends to be lower than for the other age groups, presumably because of the school summer break, as indicated in Figure 4. Conversely, at the start of the wave, the base reproduction numbers for the middle-aged groups and the elderly tend to be higher. Therefore, prioritizing these age groups is a more effective measure for preventing infection spread at that point in time.

Severe cases

As shown in Figure 5, the `ElderlyFirst` strategy leads to the lowest cumulative severe-case incidence; it performs similar to (third wave) or better than (fourth wave) the `Factual` strategy. The `Uniform` and `YoungFirst` strategies lead to the highest cumulative severe-case incidence. Which one of these two leads to the highest severe-case incidence depends on the wave and the assumed contact mixing factor (supplement C).

Figure 6 shows the trade-offs made between age groups in terms of severe cases under the different strategies. As expected, the younger age groups benefit most from the `YoungFirst` strategy, and older age groups experience the lowest severe-case incidence under the `ElderlyFirst` strategy.

The severe-case risk is lowest for the youngest age groups, as shown in Figure 3D. Hence, prioritizing the younger age groups leaves the older age groups (which are at higher risk) less protected against severe cases. While the `YoungFirst` strategy reduces the severe-case incidence in the youngest age group, this reduction is far outweighed by the increase in most other age groups. We find the opposite effect for the `ElderlyFirst` scenario; we have a reduction of severe-case incidence in the elderly and an increase in the younger age groups.

Our results suggest that, under the `ElderlyFirst` strategy, the cumulative severe-case incidence could have been reduced

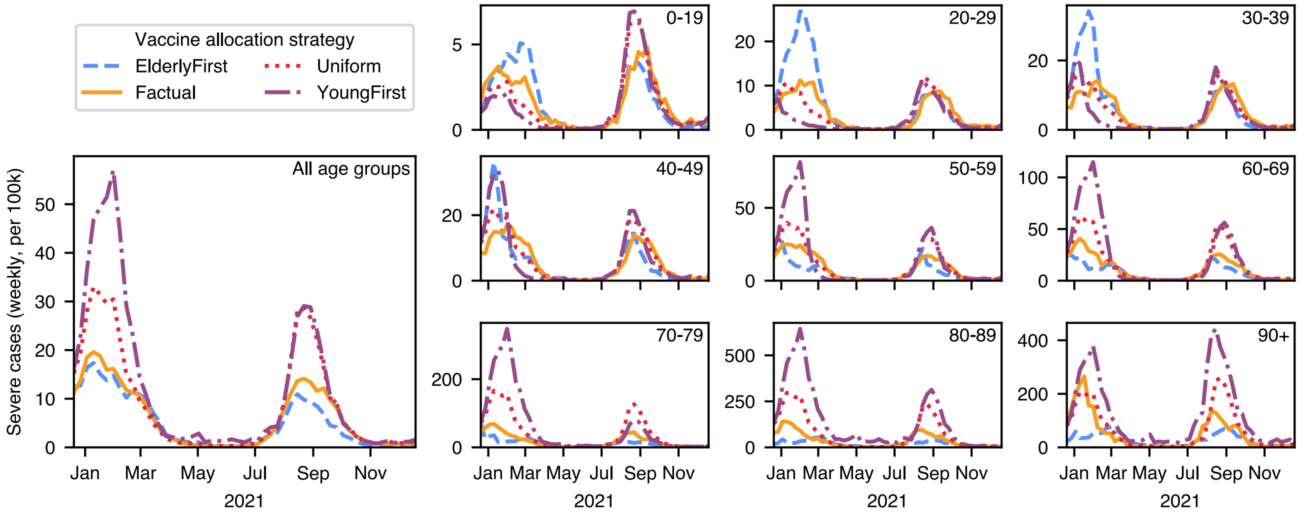

Figure 6. Expected weekly number of severe-case incidence for the entire population and in each age group for the factual and counterfactual vaccine allocation strategies

The right panels show the trade-offs in severe-case incidence between age groups under different vaccine allocation strategies. The ticks on the x axis indicate the first day of the respective month. The outcome of the `factual` strategy is taken from data,[22] while the other vaccine allocation strategies are simulated.

had this strategy been implemented in Israel (177 vs. 184 per 100,000 and 84 vs. 126 per 100,000 in the third and fourth waves, respectively). However, our counterfactual vaccine allocation strategies make some simplifying assumptions that are difficult to implement in practice. We assume that it is possible to vaccinate all willing patients of an age group before moving on to the next age group without delays. In practice, this is difficult to accomplish, in particular given that it may be harder to reach the elderly. Therefore, no realistic vaccine allocation strategy can be as strict as the protocol followed in `ElderlyFirst` and will have some overlap between age groups. Hence, from our counterfactual results and these observations, we conclude that the `Factual` strategy may have been close to optimal.

Impact of increasing vaccine uptake

We simulate the effect of increasing vaccine uptake in a single age group. Limited vaccine uptake is a factor that prolongs the necessity of non-pharmaceutical interventions.[3] One possible intervention governments have at their disposal is to encourage vaccinations through advertisement campaigns. Such campaigns can be targeted at specific age groups by choosing the channel over which the campaign is broadcast. Our method allows us to estimate the impact of increasing vaccine uptake on the expected incidence of infections and severe outcomes. We simulate the effect of increasing the vaccine uptake rate within one single given age group by administering an additional $N = 55,746$ doses (0.6% of the total population) to that age group. All other age groups follow the simulated factual vaccine allocation strategy. The additional doses are spread over the entire time period by scaling the weekly administered doses by a constant factor.

Increasing vaccine uptake in the oldest age groups is most effective in decreasing the severe-case incidence in the total population, as shown in Figure 7. On the other hand, increasing vaccine uptake in the middle-aged groups is most effective in decreasing the infection incidence in the total population.

There are two effects of an increase in vaccine uptake. First, there is a larger number of individuals who are better protected against infection and severe cases through vaccine-induced immunity. This effect can be explained by the risk factors shown in Figure 3D; by increasing the vaccine uptake rate in a given age group, we effectively move an additional part of this population from vaccination status 0 (unvaccinated) to 3 (boostered). We find the largest effect for the elderly because they have the largest absolute difference in risk factors between vaccination states 0 and 3. Second, there is a population-level effect; by influencing the infection spread, the total number of infections is reduced (Figure 7, left). Because the base reproduction number tends to be higher for the middle-aged groups (Figure 4, right), they have a larger impact on the number of infections when their vaccine uptake rate is increased. However, when considering the severe-case incidence, this effect on infections is not large enough to outweigh the differences in risk factors, as shown in Figure 7 (right). In summary, even when taking into account the effect on infection dynamics, it would have been most beneficial to increase the vaccine uptake rate in the elderly.

Simulating other disease types

To investigate whether one can generalize the recommendation to first vaccinate age groups with the highest severe-case risk, we simulate different types of diseases by adopting other risk factors. The explicit factorization of the severity mechanism (2) allows us to dissect the different contributing factors that determine the expected severe-case incidence. One of those factors is the age- and vaccination-dependent risk profile of COVID-19. Here, we showcase the ability of our model to be adapted to different diseases in fictional but plausible scenarios. We compare three disease types, shown in Figure 8 (left). (1)

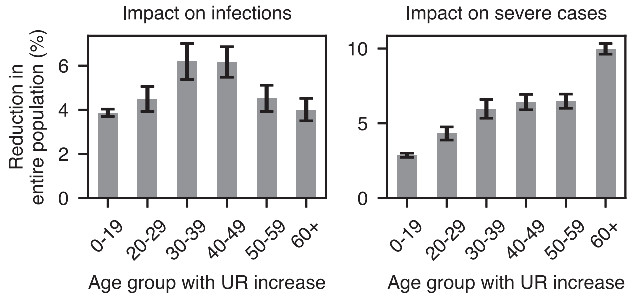

Figure 7. Impact of increasing vaccine uptake rate (UR) in a given age group

In each scenario, the vaccine UR is increased in a given age group by a fixed number corresponding to 0.6% of the population being motivated to get vaccinated. We assume that the change comes from originally unvaccinated individuals who are persuaded to receive three doses. The plots show the impact on cumulative infections (left) and severe cases (right) in the entire population, not just in the age group in which the UR was increased. We group the three oldest age groups into one to increase the total number of additional doses we can give to an age group (the oldest age groups have the smallest share of the population). We consider cases from December 20, 2020 to December 25, 2021. While the middle-aged groups have a larger impact on infection dynamics, this effect is outweighed by the higher severe-case risk in the 60 + age group when considering the impact on severe cases. The whiskers show the 95% credible intervals.

COVID-19 (Figure 3). (2) Spanish flu. We use age-specific excess respiratory death rates associated with the Spanish flu pandemic in Kentucky (1918–1919)[30] as an approximation for the risk factors $g(0, A)$ for the Spanish flu. To obtain the other risk factors $g(V>0, A)$, we assume a constant vaccine efficacy for all age groups. (3) Flat risk. This simulates a disease where all age groups have the same severe-case risk.

Lacking adjusted estimates, and for simplicity and comparability, the other factors of the severity factorization (2), f^0, $f^1_{\bar{\pi}}$, and h^V, are assumed to be the same as for COVID-19 (shown in Figure 3). The risk profiles are normalized so that the cumulative severe-case incidence is equal under the `Uniform` vaccine allocation strategy.

Besides the vaccine allocation strategies shown in Counterfactual vaccine allocation strategies, we consider two additional strategies that take into account the altered risk profiles: (1) `RiskRanked`: we prioritize age groups in descending order of the risk factors. (2) `RiskRankedReversed`: we prioritize age groups in ascending order of the risk factors. Note that, for the `flat` risk profile, these two strategies are identical to `Uniform` because all age groups have the same risk.

The factual vaccine uptake rate is influenced by the age-specific risk structure of COVID-19. Because the elderly have a higher severe-case risk, they have more incentive to get vaccinated. To remove this bias from the simulation setup, we assume a flat vaccine uptake rate of 90% willingness to receive all three vaccine doses across all age groups for the Flat and Spanish flu risk profiles.

There are no differences between diseases regarding the cumulative infection incidence under the four initial vaccine allocation strategies: `Factual`, `Uniform`, `ElderlyFirst`, and `YoungFirst`. Of those, the strategies that prioritize young and middle-aged groups are most effective at preventing infection spread during the third wave, as discussed in Counterfactual vaccine allocation strategies. For COVID-19, the `RiskRanked` and `RiskRankedReversed` strategies are most similar to the `Factual` and `YoungFirst` strategies, respectively, and lead to similar infection incidence. For the flat risk, `RiskRanked` and `RiskRankedReversed` are identical to the `Uniform` strategy and lead to the same infection outcome. For the Spanish flu, the most effective strategy for preventing infection spread is `RiskRanked` because it prioritizes age groups 20–29 and 30–39, which tend to have the highest base reproduction numbers during the third wave (Figure 4); conversely, `RiskRankedReversed` is the least effective.

For COVID-19, the severe-case incidence is lowest for strategies that prioritize high-risk age groups and lowest for strategies that do the reverse, as discussed in Counterfactual vaccine allocation strategies. The same can be said for the Spanish flu; however, the adversarial strategy `RiskRankedReversed` leads to even worse outcomes. For COVID-19, younger and middle-aged groups have high base reproduction numbers during the third wave, and the elderly have high severe-case risk. For the Spanish flu, however, the age groups with high severe-case risk and high base reproduction numbers are the same (20–29 and 30–39). Therefore, the `RiskRankedReversed` strategy is adversarial in two ways: it leads to a high infection and severe-case incidence.

In summary, this simulation shows that, even when taking into account infection dynamics, following a strategy where people most at risk are vaccinated first leads to the least amount of severe cases. However, we remark that the difference in severe cases is not as large as one could assume based on the difference in infection-fatality ratio alone. For COVID-19, a difference of 20 years increases fatality by a factor of 10, but different vaccination strategies only differ at maximum by a factor of 4. The protection against infection granted by the vaccines and the subsequent contribution to mitigation of the epidemic waves decreases the differences of the outcomes of the different strategies.

Impact of immunity waning

We investigate the influence of immunity waning on infection and severe-case incidence. We compare three settings for the timescale at which immunity weakens. (1) Regular: we use the waning function derived from the results reported in Tartof et al.[23] (2) No waning: we assume the vaccine efficacy against infection stays constant at the maximum. (3) Fast: the waning is 25% faster than regular; i.e., it takes 25% less time until vaccine efficacy is halved.

The other factors of the severity factorization (2), f^0, $f^1_{\bar{\pi}}$, and g, are assumed to be the same as before (Figure 3). For each setting of the waning function, we run the four vaccine allocation strategies discussed in Counterfactual vaccine allocation strategies.

The cumulative infection incidence increases as the speed of waning increases, as shown in Figure 9B. This difference is mainly driven by the behavior of the fourth wave, where the average times since the last dose are highest. The different strategies perform similarly; the differences in effectiveness of preventing infection spread between strategies is small compared with the differences between waning profiles. The severe-case incidence follows the same pattern; the faster the waning, the higher the severe-case incidence (Figure 9C).

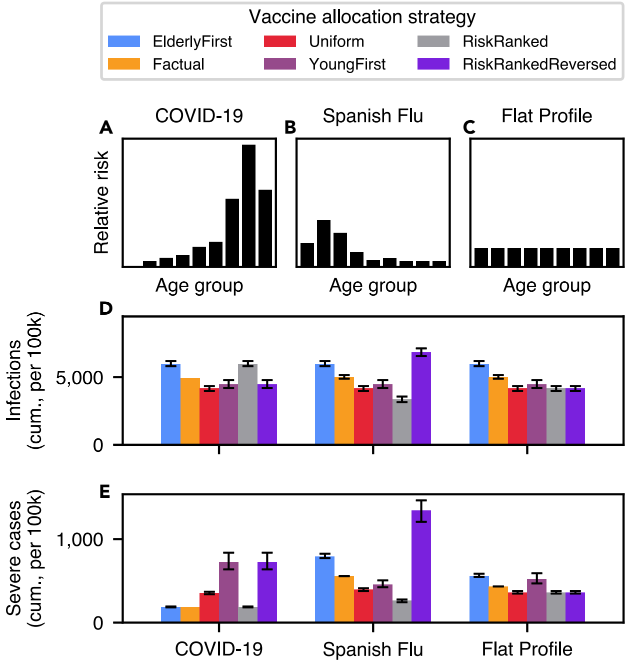

Figure 8. Comparison of risk profiles or disease types

(A–C) Age-specific risk factors for the unvaccinated $g(V=0, A)$ for three considered disease types. Vaccine efficacy is assumed to be independent of age and the same across all disease types. The risk factors are normalized across disease types to lead to the same number of severe cases under the `Uniform` vaccine allocation strategy.

(D and E) Cumulative incidences of infections (D) and severe cases (E) under different vaccine allocation strategies between December 20, 2020 and April 11, 2021 (third wave). The whiskers show the 95% credible intervals. The outcome of the `Factual` strategy for COVID-19 is taken from data;[22] all other scenarios are simulated.

These results highlight the influence of immunity waning on infection dynamics. A relatively moderate reduction by 25% of the waning timescale leads to an increase in infection and severe-case incidence by a factor 3 over the time span of a year. This illustrates two points. First, for accurate infection dynamics predictions, it is crucial to have a good estimate of the waning curve. Second, it shows the importance of regular vaccinations, counteracting the waning effect.

DISCUSSION

We have built a model of how the severe-case probability depends on relevant factors such as age and vaccination status. This model uses data, simulation, and prior knowledge in a modular fashion and combines parameter inference and prediction. We used data on infections and severe cases to retrospectively evaluate different strategies in a realistic setting and explore counterfactual scenarios. We were able to simulate the impact of increasing vaccine uptake by age group. The modular structure of our approach also allowed us to evaluate the behavior of different types of diseases and the role of immunity waning.

Previous work that retrospectively evaluated vaccination campaigns focused mainly on estimating the overall success in reducing infections, hospitalizations, and deaths by modeling counterfactual scenarios with fewer or no vaccinations.[31,32] In addition, there have been simulation studies conducted before the start of the vaccination campaign that, similar to our work, evaluate different age-dependent vaccine allocation strategies.[17,33–35] The forward-looking studies find that, in general, the middle-aged groups have the biggest impact on infection incidence (see, e.g., Figure 1 in Bubar et al.[33] for demographics corresponding to the United States), which is in agreement with our findings (Figure 7, left). However, in these studies, such differences in contribution to spread stem from assumptions on the contact matrix[17,33,35] or prior knowledge of susceptibility,[17,33,34] whereas in our method, we estimate the age-dependent reproduction numbers directly from infection data. The agreement suggests that both approaches lead to qualitatively similar results. Other studies investigating age-dependent transmission inferred from observed cases[36] or seropositivity data[37] also find that middle-aged groups have the largest impact on transmission. In the present work, by retrospectively taking into account observed infection data, we were additionally able to show that the timing of the vaccination campaign relative to non-pharmaceutical interventions is crucial. In the third wave, for instance, with partially open schools in Israel, vaccinating the young would have been most effective at preventing infections, whereas, in the fourth wave, with school holidays during the start of the wave, this strategy would have been among the least effective (Figure 5). Nevertheless, when it comes to minimizing severe cases, all studies agree that (under realistic parameter settings[34]) prioritizing the elderly is most effective because of the large difference in the infection-fatality ratio[17,33,34] (Figures 5 and 7, right). This tradeoff between direct protection of at-risk groups (direct protection) and protection through vaccinating those contributing most to spreading (indirect protection) has also been discussed in Markovič et al.[7]

While we were able to include many factors relevant to severe cases and infections, our method has some limitations. Our approach relies on assumptions, which are only approximately correct and difficult to test in practice. We assume causal sufficiency[8] for the variables in our causal model, which rules out confounding between the variables on which we intervene (vaccination times) and the outcome (severe cases). However, we do expect some confounding in practice; at-risk groups like healthcare workers or patients with pre-existing conditions may have a higher incentive to get vaccinated.[7,35,38] This could break the assumption of homogeneous subgroups based on age, vaccination status, and waning time and lead to overestimated risk factors for the vaccinated: $g(V>0, A)$.

In our study, we quantify the number of infections and severe cases, where we weigh all cases equally across age groups. However, to more accurately quantify the impact on society, we could consider more factors, such as differences in predisposition to long COVID.[39] Differences in length and intensity of suffering could also be captured by metrics such as quality-adjusted life years.[40] However, such metrics are difficult to estimate in practice.

In the present work, we do not explicitly model behavioral or policy responses in our counterfactual scenarios. However, high infection incidence increase perceived risk in the population and prompt voluntary health-protective behavior.[41] Governments also react to changes in infection incidence by introducing or relaxing non-pharmaceutical interventions. Both effects tend to

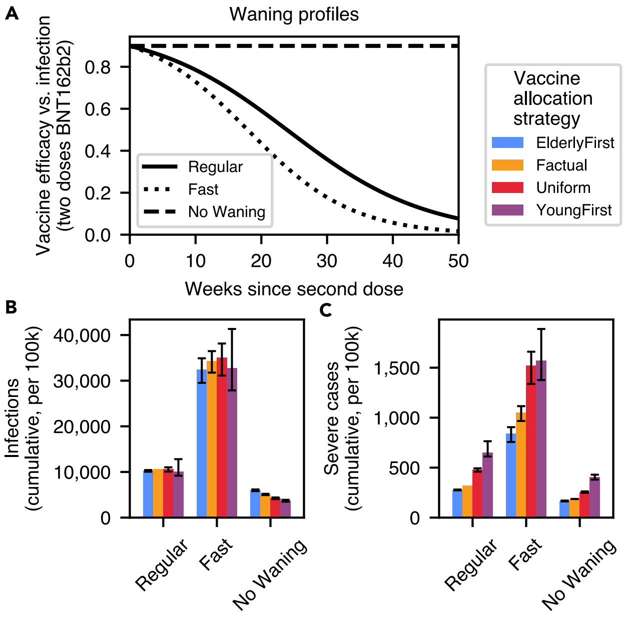

Figure 9. Comparison of waning timescales

(A) The considered immunity waning timescales. In the fast waning scenario, we assume it takes 25% less time until vaccine efficacy against infection is halved compared with the timescale reported in Tartof et al.[23]

(B and C) The cumulative infection (B) and severe-case (C) incidences under different vaccine allocation strategies for each waning timescale. We sum all cases from December 20, 2020 to December 25, 2021. The whiskers show the 95% credible intervals. The outcome of the `Factual` strategy for regular waning is taken from data;[22] all other scenarios are simulated.

reduce (and increase, respectively) the effective reproduction number during high (and low, respectively) incidence periods. Therefore, we expect our infection and severe-case incidence to be overestimated for high-incidence periods and underestimated for low-incidence periods. Our results should thus be interpreted as counterfactual vaccine allocation scenarios while keeping behavioral and government responses fixed.

We are also neglecting possible selection bias through differences in testing frequencies between age groups. School children may be tested more frequently than other age groups during school weeks, which could lead to overestimating their contribution to infection spread and, consequently, the effect of vaccinating children.

Besides approaching the problem of evaluating counterfactual vaccine allocation strategies, this study illustrates a more general problem in causal inference. Causal models typically require joint observations of all relevant variables to evaluate counterfactual statements. However, in realistic settings, data are often a limiting factor. In the present study, two crucial parts were not observed: waning times of the severely ill and latent factors related to infection dynamics (such as the base reproduction numbers). By assuming a factorization for the severity mechanism (2) and incorporating an SEIR-like model together with literature estimates for waning, we were able to address these limitations. In our case, we leveraged domain expertise to merge the different sources of knowledge—namely, data, simulation, and literature estimates—into one model. Some first steps in this direction have been taken,[42,43] but it is still an open question how to merge information from different sources or datasets into a single causal model without strong assumptions stemming from domain knowledge.

Through our simulation-assisted causal model, we show how interactions between different elements of a pandemic, such as vaccinations, immunity waning, age-dependent infection spread, and risk can be effectively captured. While we have applied our method retrospectively, with parameter inference on observational data, this does not limit its applicability.

While the overall model is unlikely to be valid for other pandemics, sub-modules may be transferable.[44] Moreover, each of the sub-modules can be replaced with appropriate assumptions on parts of the system that are still unknown, as shown in Simulating other disease types and Impact of immunity waning. For example, when COVID-19 vaccines were approved initially, it was not yet clear how long immunity against infection or a severe course would last. Thus, besides informing the rollout of COVID-19 vaccination campaigns, we hope that our method can help in future pandemics where the relevant factors may not yet be jointly measured or known from the literature.

EXPERIMENTAL PROCEDURES

Resource availability

Lead contact

Further information and requests for resources and materials should be directed to and will be fulfilled by the lead contact, Armin Kekić (armin.kekic@tuebingen.mpg.de).

Materials availability

This study did not generate new unique materials.

Data and code availability

The epidemiological data that support the findings of this study are available from the Ministry of Health Israel[22] (https://data.gov.il/dataset/covid-19). The population data used for estimating the age distribution in Israel are available from the United Nations World Population Prospects 2019[45] (https://population.un.org/wpp). The source code is available at https://github.com/akekic/covid-vaccine-evaluation [46] and contains copies of all used data sources.

Target function

The target function $s(\tilde{\pi} = \tilde{P}(T_1, T_2, T_3 | A))$ describes the relationship between the counterfactual vaccination policy $\tilde{\pi}$ and the resulting expected number of severe cases. It can be written as

$$s(\tilde{\pi} = \tilde{P}(T_1, T_2, T_3 | A)) = MD\, \mathbb{E}[S | \mathrm{do}(T_1, T_2, T_3 \sim \tilde{P}(T_1, T_2, T_3 | A))] \quad \text{(Equation 4)}$$

$$\begin{aligned} &= D \sum_a P(a) \sum_{t=1}^{M} f^0(t) f^1_{\tilde{\pi}}(a,t) \\ &\times \left[\sum_{t_1 = t+1}^{M+1} \sum_{t_2 = t+1}^{M+1} \sum_{t_3 = t+1}^{M+1} \tilde{P}(t_1, t_2, t_3 | a) g(0, a) \right. \\ &+ \sum_{t_1 = 1}^{t} \sum_{t_2 = t+1}^{M+1} \sum_{t_3 = t+1}^{M+1} \tilde{P}(t_1, t_2, t_3 | a) g(1, a) h^1(t - t_1) \\ &+ \sum_{t_1 = 1}^{t} \sum_{t_2 = 1}^{t} \sum_{t_3 = t+1}^{M+1} \tilde{P}(t_1, t_2, t_3 | a) g(2, a) h^2(t - t_2) \\ &+ \left. \sum_{t_1 = 1}^{t} \sum_{t_2 = 1}^{t} \sum_{t_3 = 1}^{t} \tilde{P}(t_1, t_2, t_3 | a) g(3, a) h^3(t - t_3) \right] \end{aligned} \quad \text{(Equation 5)}$$

where D is the total population. For notational convenience, we treat individuals who have not received a certain dose by setting the respective time of vaccination to $t_i = M + 1$. The full derivation is shown in supplement A.2.

Estimating the severity mechanism factors

Risk factors for the unvaccinated

Estimating the factorization (2) is ill posed because the overall scale of the factors g, h^V and f^0 is not well defined. We can double f^0 and halve g and end up with the same value for the severity mechanism. We remove this ambiguity by setting

$$g(0,a^*) = 1 \qquad \text{(Equation 6)}$$

for some age group a^*. By common convention, we choose the age group $60-69$ as the reference group so that all other risk factors are relative to $g(0,a^*)$. Note that there is no waning for $V = 0$, hence $P(S = 1|V = 0, A, T, W) = P(S = 1|V = 0, A, T)$. We can then estimate the other risk factors of the unvaccinated by

$$\hat{g}(0,a) = \frac{\mathbb{E}_T[P(S=1|V=0,A=a,T)]}{\mathbb{E}_T[P(S=1|V=0,A=a^*,T)]}. \qquad \text{(Equation 7)}$$

Immunity waning curve

The vaccine efficacy against infection as a function of time since administration of the second dose of the BioNTech vaccine is reported in Tartof et al.[23] for discrete time periods up to 6 months. To these data, we fit a logistic curve that tends toward zero efficacy as time increases. For the waning curves after 1 and 3 doses, we assume the same functional relationship as for the second dose but scale the function so that, under full protection, the efficacy is 75% and 95%, respectively (efficacy under full protection is around 90% after two doses).

We can use this to derive the waning function $h^V(W)$. First observe that the severe-case probability can be separated into two processes:

$$P(S=1|V,A,T,W) = \underbrace{P(I=1|V,A,T,W)}_{(a)}\underbrace{P(S=1|V,A,I=1)}_{(b)} \qquad \text{(Equation 8)}$$

(a) the probability of being infected and (b) the probability of developing a severe case when infected. We assume that the probability of having a severe case when infected only depends on V and A and that the immunity against severe courses does not significantly wane over time, as reported in Tartof et al.[23] Now, let $\mathrm{VE}^v(w)$ be the vaccine efficacy w weeks after receiving the vth dose:

$$\mathrm{VE}^v(w) = 1 - \frac{P(I=1|V=v,A=a,T,W=w)}{P(I=1|V=0,A=a,T,W=w)} \qquad \text{(Equation 9)}$$

where we assume that the efficacy against infection is the same for all age groups and constant over time T. Then note, using Equation 8,

$$\frac{P(S=1|V=v,a,t,w)}{P(S=1|V=0,a,t,w)} = \frac{P(S=1|V=v,a,I=1)}{P(S=1|V=0,a,I=1)}\underbrace{\frac{P(I=1|V=v,a,t,w)}{P(I=1|V=0,a,t,w)}}_{1-\mathrm{VE}^v(w)} \qquad \text{(Equation 10)}$$

$$= \frac{f^0(t)g(v,a)h^v(w)}{f^0(t)g(0,a)} = \frac{g(v,a)}{g(0,a)}h^v(w) \qquad \text{(Equation 11)}$$

$$\Rightarrow h^v(w) = \frac{g(0,a)}{g(v,a)}\frac{P(S=1|V=v,a,I=1)}{P(S=1|V=0,a,I=1)}(1-\mathrm{VE}^v(w)). \qquad \text{(Equation 12)}$$

Note that, because we are considering the factual vaccine allocation strategy π, the correction factor is $f_\pi^1(A,T) = 1$. By definition, we have $h^v(0) = 1$, and combining the above expression for $W = w \geq 0$ and $W = 0$, we get

$$\frac{h^v(w)}{h^v(0)} = \frac{1-\mathrm{VE}^v(w)}{1-\mathrm{VE}^v(0)} \Rightarrow h^v(w) = \frac{1-\mathrm{VE}^v(w)}{1-\mathrm{VE}^v(0)}. \qquad \text{(Equation 13)}$$

Risk factors for the vaccinated

In our data,[22] we can only observe $P(S = 1|V, A, T)$ because we do not have data on severe outcomes as a function of the time since the last dose W. However, we do have data on the distribution of times since the last dose was received $P(W|V,A,T)$. This allows us to use the waning function (13) to estimate the risk factors for $V = v > 0$ since

$$P(S=1|V=v,a,t) = \sum_w P(S=1|V=v,a,t,w)P(w|V=v,a,t) \qquad \text{(Equation 14)}$$

$$= f^0(t)g(v,a)\sum_w h^v(w)P(w|V=v,a,t). \qquad \text{(Equation 15)}$$

This motivates the following estimator:

$$\hat{g}(v,a) = \frac{\mathbb{E}_T\left[\frac{P(S=1|V=v,A=a,T)}{\mathbb{E}_{W|V=v,A=a,T}[h^v(W)]}\right]}{\mathbb{E}_T[P(S=1|V=0,A=a,T)]}\hat{g}(0,a), \qquad \text{(Equation 16)}$$

i.e., we correct for the waning that occurred in the population to estimate the risk factor under full immunity.

Time dependence

After correcting for immunity waning and differences in risk factors, we can estimate the overall time dependence:

$$\hat{f}(t) = \mathbb{E}_{V,A|T=t}\left[\frac{P(S=1|V,A,T=t)}{\hat{g}(V,A)\mathbb{E}_{W|V,A,T=t}[h^V(W)]}\right]. \qquad \text{(Equation 17)}$$

Infection dynamics correction factor

For the estimation of $f_{\tilde{\pi}}^1(A, T)$, we consider the following: let $P_\pi(S = 1|V, A, T, W)$ be the severity mechanism under the observed vaccine allocation strategy π and $P_{\tilde{\pi}}(S = 1|V, A, T, W)$ under the post-intervention vaccine allocation strategy $\tilde{\pi}$. Then, using Equation 8,

$$\frac{P_{\tilde{\pi}}(S=1|V,A,T,W)}{P_\pi(S=1|V,A,T,W)} = \frac{P_{\tilde{\pi}}(I=1|V,A,T,W)}{P_\pi(I=1|V,A,T,W)}\frac{P(S=1|V,A,I=1)}{P(S=1|V,A,I=1)} = \frac{P_{\tilde{\pi}}(I=1|V,A,T,W)}{P_\pi(I=1|V,A,T,W)} \qquad \text{(Equation 18)}$$

where we have used that the process of going from infected to severely ill (b) does not depend on the vaccine allocation strategy. From the factorization of the severity mechanism (2) and using $f_\pi^1(A,T) = 1$, it follows that

$$\frac{P_{\tilde{\pi}}(S=1|V,A,T,W)}{P_\pi(S=1|V,A,T,W)} = f_{\tilde{\pi}}^1(A,T). \qquad \text{(Equation 19)}$$

The assumption that $f_{\tilde{\pi}}^1$ only depends on A and T means we assume that the change in infection probability because of the infection dynamics is independent of V and W. Hence, the correction factor is the relative change in weekly infection probability for each age group under the counterfactual vaccine allocation strategy:

$$f_{\tilde{\pi}}^1(A,T) = \frac{P_{\tilde{\pi}}(I=1|V,A,T,W)}{P_\pi(I=1|V,A,T,W)}. \qquad \text{(Equation 20)}$$

Modeling infection dynamics

To estimate the effect of changing the vaccine allocation strategy on the infection dynamics, we first infer the parameters of a Bayesian SEIR-like model to describe infections $P_\pi(I = 1|V, A, T, W)$ under the observed policy π. We then rerun the model with the inferred reproduction numbers under the counterfactual strategy $\tilde{\pi}$ to obtain an estimate of $P_{\tilde{\pi}}(I = 1|V, A, T, W)$. The correction factor $f_{\tilde{\pi}}^1(A, T)$ is given by the ratio of these two infection probabilities (Equation 3).

The SEIR-like dynamics

In our model, each age group a has its own compartment, and the dynamics follow a discrete renewal process[1,47] determined by the effective reproduction number $R_{\text{eff},a}(t_{\text{day}})$. These dynamics are discretized with a one-day step to allow enough resolution to model the generation interval. The data are only available on a weekly basis, which will require us later to sum the cases over 1 week. We fit one reproduction number per age group. The infections from one age group to another are encoded by a contact matrix $\boldsymbol{C}$. The latent period is modeled by a kernel $g(\tau)$, which is normalized to 1:

$$E_a(t_{\text{day}}) = \sum_{a'=1}^{9}\sqrt{R_{\text{eff},a}(t_{\text{day}})}C_{a,a'}\sqrt{R_{\text{eff},a'}(t_{\text{day}})}\sum_{\tau=0}^{10}E_{a'}(t_{\text{day}}-1-\tau)g(\tau) + h_a(t_{\text{day}}), \qquad \text{(Equation 21)}$$

$$S_a(t_{day}) = S_a(t_{day}-1) - E_a(t_{day}-1), \quad \text{(Equation 22)}$$

$$g(\tau) = \text{Gamma}(\tau; \mu = 4, \sigma = 1.5). \quad \text{(Equation 23)}$$

$E_a(t_{day})$ is the number of newly exposed people on day t_{day}, who will later become infectious; it is implicitly modeled by the generation interval kernel g. We assume here a mean generation interval of 4 days.[24,25] $S_a(t_{day})$ is the number of susceptible people, and $h_a(t_{day})$ is an external influx (see below).

We assume that changes in the reproduction number symmetrically affect the infectiousness and infectability. This is achieved by multiplying the square root of the reproduction number to the contact matrix from both sides in Equation 21.

The contact matrix

While the effective reproduction numbers describe the overall level of infection-transmitting contacts for each age group, the contact matrix models how these contacts are distributed between age groups. In other words, the former describes how many infection-transmitting contacts a given age groups has, and the latter describes how often these contacts are among their own or other age groups. The following consistency requirements need to be satisfied by the contact matrix.

- For unit effective reproduction numbers for a given time t_{day}, $R_{eff,a}(t_{day}) = 1\,\forall a$, the total number of exposed should stay constant. This is achieved by column-wise normalized matrices so that $1\sum_{a'} C_{a,a'} = 1$.
- For constant unit effective reproduction numbers, $R_{eff,a}(t_{day}) = 1\,\forall a, t_{day}$, the limit distribution of cases over the age groups should be proportional to the population share. This corresponds to the eigenvector for the largest eigenvalue of C being proportional to the vector of the population share in each age group $\vec{\rho}^{\,T} = (\rho_1, \rho_2, \ldots) = \left(\frac{D^{(A=1)}}{D}, \frac{D^{(A=2)}}{D}, \ldots\right)$, where $D^{(A=a)}$ is the population of age group a, and $D = \sum_a D^{(A=a)}$ is the total population. In the same setting, the total number of cases should not diverge or tend to zero. This can be ensured by contact matrices with the largest eigenvalue 1.

There are two extreme cases for mixing patterns under these constraints.

1. There are no contacts between age groups. In this case, the contact matrix would simply be the identity matrix: C = 1.
2. The contact between different age groups is the same as within age groups: all-to-all connectivity.

In this case, the contact matrix would be $\boldsymbol{C} = \vec{\rho} \cdot \vec{1}^{\,T}$.

The reality lies somewhere between these two cases. A reasonable interpolation between these extremes should ensure that the largest eigenvector of the contact matrix stays $\vec{\rho}$ and that the largest eigenvalue is 1. These requirements are met by the following matrix:

$$\boldsymbol{C} = (1-\gamma)1 + \gamma\vec{\rho}\cdot\vec{1}^{\,T} = \begin{pmatrix} \gamma\rho_1 + (1-\gamma) & \gamma\rho_1 & \gamma\rho_1 & \cdots \\ \gamma\rho_2 & \gamma\rho_2 + (1-\gamma) & \gamma\rho_2 & \cdots \\ \gamma\rho_3 & \gamma\rho_3 & \gamma\rho_3 + (1-\gamma) & \cdots \\ \vdots & \vdots & \vdots & \ddots \end{pmatrix}. \quad \text{(Equation 24)}$$

The parameter γ determines the contact mixing between age groups; at 1 we have all-to-all connectivity, and at 0 there are no contacts between age groups.[48,49]

Because one of the goals of this study is to understand age dependence, we have designed our infection dynamics simulation so that it can infer age-dependent reproduction numbers directly from data rather than making assumptions about age-dependent effects. Diary-based estimates of contacts strength[50,51] are not suitable for our purposes because they do not meet the requirements outlined above. In particular, they contain distributions of contacts between age groups and overall levels of contacts, which we try to estimate over time. This leads the leading eigenvectors of these matrices to be non-uniform (after accounting for differences in population share).

The external influx

To account for some infections occurring because of infected travelers entering Israel, we add a random number of infections distributed over each week:

$$h_a(t_{day}) = h_a^*(t = k_{week}(t_{day}))/7, \quad \text{(Equation 25)}$$

$$h_a^*(t) \sim \text{Weibull}\left(\lambda = 0.1\cdot\frac{\text{pop}_a}{10^6}, k = 0.3\right) \quad \forall a, \forall t, \quad \text{(Equation 26)}$$

where t is indexing the weeks included in our analysis, and $k_{week}(t_{day})$ is mapping a day t_{day} to the corresponding week t. We chose a Weibull distribution because the long tails allow the occurrence of mass spreading events. The parameters of the Weibull distribution are chosen so that, on average, 0.1 infections per million inhabitants per day occur from external influx. This is about a fifth of the lowest incidence during the analysis period. The median of the distribution is only at 0.003 infections per million inhabitants per day because of the long tails of the distribution.

The effective reproduction number

The effective reproduction number $R_{eff,a}(t_{day})$ depends on (1) the base reproduction number $R_{base,a}(t_{day})$, which encodes the amount of social distancing at time t_{day}, and (2) the infectability term $\text{Infectability}_a(t_{day})$, which encodes the acquired immunity of the susceptible population of age group a:

$$R_{eff,a}(t_{day}) = R_{base,a}(t_{day})\cdot\text{Infectability}_a(t_{day}). \quad \text{(Equation 27)}$$

Infectability

The Infectability_a is the fraction of reduced spread because of acquired immunity in age group a. It is 1 in a completely non-immune population and reduces with vaccination. Concretely, it is modeled as:‘

$$\begin{aligned}\text{Infectability}_a(t_{day}) = \text{Unv}_a(t) &+ \text{Vacc}_a^1(t)\left(1 - \mu_a^1 W_{eff,a}^1(t)\right) \\ &+ \text{Vacc}_a^2(t)\left(1 - \mu_a^2 W_{eff,a}^2(t)\right) \\ &+ \text{Vacc}_a^3(t)\left(1 - \mu_a^3 W_{eff,a}^3(t)\right)\end{aligned} \quad \text{(Equation 28)}$$

$$\text{with } t = k_{week}(t_{day}), \quad \text{(Equation 29)}$$

where $\text{Unv}_a(t)$, $\text{Vacc}_a^1(t)$, $\text{Vacc}_a^2(t)$, and $\text{Vacc}_a^3(t)$ are the fractions of unvaccinated and once, twice, and three times vaccinated, respectively, for each age group. Here $t = k_{week}(t_{day})$ is the week corresponding to the day t_{day}. μ denotes the corresponding protection against infection ($0 < \mu < 1$, and $\mu = 0$ corresponds to no protection). We assume that the protection is 70%, 90%, and 95% directly after the first, second, and third dose, respectively.[23] These vaccination fractions are modeled in a weekly manner because the data are only available on a weekly basis. $W_{eff,a}^v(t)$ denotes the effective group-wide waning of immunity for the group that has been vaccinated v times. It is 1 when the whole age group had been vaccinated a few days ago and decreases with time.

The effective waning of the group-wide immunity at time t is calculated by building an average over all individuals who have received v doses before time t weighted by their individual waning factor:

$$W_{eff,a}^v(t) = \frac{\sum_{\tau=0}^{t} \text{n\_Vacc}_{a,t}^v(\tau)\text{VE}_{norm}(t-\tau)}{\sum_{\tau=0}^{t} \text{n\_Vacc}_{a,t}^v(\tau)} \quad \text{(Equation 30)}$$

Where $\text{n\_Vacc}_{a,t}^v(\tau)$ are the newly vaccinated at time τ who have received v vaccinations by time τ, and $\text{VE}_{norm}(w) = \frac{\text{VE}(w)}{\text{VE}(0)}$ is the normalized vaccine efficacy w weeks after the last dose.[23] $\text{n\_Vacc}_{a,t}^v(\tau)$ and $\text{Vacc}_a^v(t)$ are obtained from published data from Israel.[22]

The base reproduction number

The base reproduction number $R_{base,a}(t_{day})$ is assumed to be a slowly changing factor as a function of time. It is modeled as a superposition of logistic change points $\gamma(t_{day})$ every 21 days, which are parameterized by the transient length of the change points l, the date of the change point d, and the effect of the change point $\Delta\gamma^*$. The subscript n denotes the discrete enumeration of the change points:

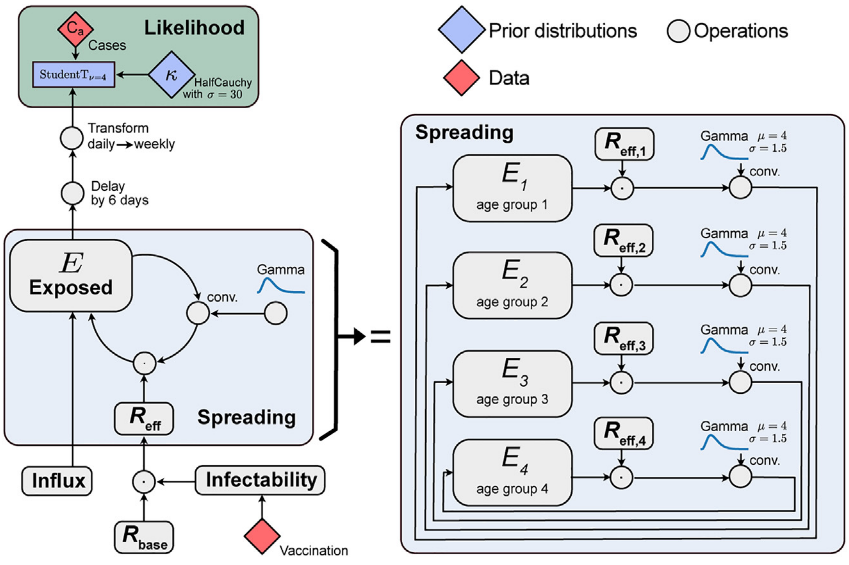

Figure 10. Overview of the infection dynamics model

The dynamics model infers the effective R_{eff} and base reproduction number as a function of time given the factual vaccine allocation strategy and the number of observed cases C_a. After inference, the model is used to predict the number of cases under a counterfactual strategy. The right side corresponds to Equation 21. Only 4 of the 9 age groups are shown here.

$$R_{\text{base},a}(t_{\text{day}}) = R_{0,a}\exp\left(\sum_n \gamma_n(t_{\text{day}})\right) \quad \text{(Equation 31)}$$

$$R_{0,a} \sim \text{LogNormal}(\mu = 1, \sigma = 1) \quad \forall a \quad \text{(Equation 32)}$$

$$\gamma_{n,a}(t_{\text{day}}) = \frac{1}{1 + e^{-4/l_{n,a}\cdot(t - d_{n,a})}}\cdot\Delta\gamma_{n,a} \quad \text{(Equation 33)}$$

$$\Delta\gamma_{n,a} \sim \mathcal{N}(\Delta\gamma_{n-1,a}, \sigma_{\Delta\gamma_a}) \quad \forall n, \forall a \quad \text{with } \Delta\gamma_{0,a} = \log R_{0,a} \quad \text{(Equation 34)}$$

$$\sigma_{\Delta\gamma,a} \sim \text{HalfCauchy}(0.5) \quad \forall a \quad \text{(Equation 35)}$$

$$l_{n,a} = \log\left(1 + \exp\left(l^{\dagger}_{n,a}\right)\right) \quad \text{(Equation 36)}$$

$$l^{\dagger}_{n,a} \sim \mathcal{N}(4, 1) \quad \forall n, \forall a \quad \text{(unit is days)} \quad \text{(Equation 37)}$$

$$d_{n,a} = 10^{\text{th}}\text{ January 2021} + 21\cdot n + \Delta d_{n,a} \quad \text{for } n = 0, \ldots, 9 \quad \text{(Equation 38)}$$

$$\Delta d_{n_a} \sim \mathcal{N}(0, 3.5) \quad \forall n, \forall a \quad \text{(unit is days)}. \quad \text{(Equation 39)}$$

The likelihood

Next, we want to define the goodness of fit of our model to the sample data. For that, the number of newly exposed people is delayed by 6 days and summed over 1 week because the case data are available on a weekly basis. The likelihood of that is modeled by a Student's t distribution, which allows some outliers because of its heavier tails compared with a normal distribution (green box in Figure 10). The error of the Student's t distribution is proportional to the square root of the number of cases, which corresponds to the scaling of the errors in a Poisson or negative binomial distribution:

$$\widehat{C}_a(t) = \sum_{t_{\text{day}}=6}^{13} E\left(k^{-1}_{\text{week}}(t) - t_{\text{day}}\right) \quad \text{(Equation 40)}$$

$$C_a(t) \sim \text{StudentT}_{\nu=4}\left(\mu = \widehat{C}_g(t), \sigma = \kappa\sqrt{\widehat{C}_a(t) + 1}\right) \quad \text{(Equation 41)}$$

$$\kappa \sim \text{HalfCauchy}(\sigma = 30). \quad \text{(Equation 42)}$$

Here $C_a(t)$ is the measured number of weekly cases in the population of age a as reported by the health authorities, whereas $\widehat{C}_g(t)$ is the modeled number of cases in week t. $k^{-1}_{\text{week}}(t)$ transforms the week t to the first day of that week.

Sampling

To estimate the parameters of the Bayesian dynamical spreading model, in particular the time-dependent base reproduction number, we use Monte Carlo sampling. In this way, we also obtain credible intervals of the parameters and not only the maximal likelihood estimate. Specifically, the sampling was performed using PyMC3[52] with the NUTS sampler,[53] which is a Hamiltonian Monte Carlo sampler.

The chains are initialized randomly. Because random initialization often leads to some chains getting stuck in local minima, we run 8 chains for 150 initialization steps and chose the 2 chains with the highest unnormalized posterior to continue tuning and sampling. We then let these chains tune for additional 500 steps and draw 500 samples. The maximum tree depth is set to 10.

Credible intervals

Infections

For all quantities related to infections, we sample from the Bayesian SEIR-like infection dynamics model to obtain samples of the posterior distribution $P_{\tilde{\pi}}(I = 1|V, A, T, W)$. For the credible interval, we take 1,000 joint samples of $P_{\tilde{\pi}}(I = 1|V, A, T, W)$ to obtain samples of the final quantity, such as the total sum of infections.

Severe cases

We compute samples for the correction factor (3) by sampling from the posterior of the infection dynamics model 1,000 times. These joint samples of the correction factor are then propagated through the target function (5) to obtain samples of the severe-case incidence.

SUPPLEMENTAL INFORMATION

Supplemental information can be found online at https://doi.org/10.1016/j.patter.2023.100739.

ACKNOWLEDGMENTS

We thank Sebastian Contreras and Simon Bauer for constructive discussions. We thank Franz Paul Spitzner for comments on visualizations. This publication was supported by the German Ministry of Science and Education (BMBF) through the Tübingen AI Center (FKZ 01IS18039A) and through RESPINOW (grant MV2021-012) and INFOXPAND (grant 031L0300A) within the MONID network; it was supported by the German Research Foundation (Deutsche Forschungsgemeinschaft, DFG) through the Cluster of Excellence "Machine Learning—New Perspectives for Science" (EXC 2064, project

390727645) and the project “Cognition of Interaction” (SFB 1528, project 454648639).

AUTHOR CONTRIBUTIONS

A.K. and J.D. wrote the software and carried out the analysis. A.K., L.G., J.v.K., and B.S. conceptualized the causal model. V.P. conceptualized the infection dynamics model. All authors contributed to development of the methodology and the manuscript draft.

DECLARATION OF INTERESTS

The authors declare no competing interests.

Received: December 6, 2022
Revised: January 16, 2023
Accepted: April 3, 2023
Published: May 3, 2023

REFERENCES

1. Brauner, J.M., Mindermann, S., Sharma, M., Johnston, D., Salvatier, J., Gavenčiak, T., Stephenson, A.B., Leech, G., Altman, G., Mikulik, V., et al. (2021). Inferring the effectiveness of government interventions against COVID-19. Science *371*, eabd9338. https://doi.org/10.1126/science.abd9338.
2. Dehning, J., Zierenberg, J., Spitzner, F.P., Wibral, M., Neto, J.P., Wilczek, M., and Priesemann, V. (2020). Inferring change points in the spread of COVID-19 reveals the effectiveness of interventions. Science *369*, eabb9789. https://doi.org/10.1126/science.abb9789.
3. Oliu-Barton, M., Pradelski, B.S.R., Algan, Y., Baker, M.G., Binagwaho, A., Dore, G.J., El-Mohandes, A., Fontanet, A., Peichl, A., Priesemann, V., et al. (2022). Elimination versus mitigation of SARS-CoV-2 in the presence of effective vaccines. Lancet Glob. Health *10*, e142–e147. https://doi.org/10.1016/S2214-109X(21)00494-0.
4. Piraveenan, M., Sawleshwarkar, S., Walsh, M., Zablotska, I., Bhattacharyya, S., Farooqui, H.H., Bhatnagar, T., Karan, A., Murhekar, M., Zodpey, S., et al. (2021). Optimal governance and implementation of vaccination programmes to contain the COVID-19 pandemic. R. Soc. Open Sci. *8*, 210429. https://doi.org/10.1098/rsos.210429.
5. O'Driscoll, M., Ribeiro Dos Santos, G., Wang, L., Cummings, D.A.T., Azman, A.S., Paireau, J., Fontanet, A., Cauchemez, S., and Salje, H. (2021). Age-specific mortality and immunity patterns of SARSCoV-2. Nature *590*, 140–145. https://doi.org/10.1038/s41586-020-2918-0.
6. Rosen, B., Waitzberg, R., and Israeli, A. (2021). Israel's rapid rollout of vaccinations for COVID-19. Isr. J. Health Policy Res. *10*, 6. https://doi.org/10.1186/s13584-021-00440-6.
7. Markovič, R., Šterk, M., Marhl, M., Perc, M., and Gosak, M. (2021). Socio-demographic and health factors drive the epidemic progression and should guide vaccination strategies for best COVID-19 containment. Results Phys. *26*, 104433. https://doi.org/10.1016/j.rinp.2021.104433.
8. Pearl, J. (2009). Causality (Cambridge University Press). https://doi.org/10.1017/CBO9780511803161.
9. Rubin, D.B. (1974). Estimating causal effects of treatments in randomized and nonrandomized studies. J. Educ. Psychol. *66*, 688–701. https://doi.org/10.1037/h0037350.
10. Fisher, R.A. (1935). The Design of Experiments (Oliver and Boyd).
11. Polack, F.P., Thomas, S.J., Kitchin, N., Absalon, J., Gurtman, A., Lockhart, S., Perez, J.L., Pérez Marc, G., Moreira, E.D., Zerbini, C., et al. (2020). Safety and efficacy of the BNT162b2 mRNA covid-19 vaccine. N. Engl. J. Med. *383*, 2603–2615. https://doi.org/10.1056/NEJMoa2034577.
12. Holland, P.W. (1986). Statistics and causal inference. J. Am. Stat. Assoc. *81*, 945–960. https://doi.org/10.1080/01621459.1986.10478354.
13. Shalit, U., Johansson, F.D., and Sontag, D. (2017). Estimating individual treatment effect: generalization bounds and algorithms. In International Conference on Machine Learning, pp. 3076–3085.
14. Helbing, D., Brockmann, D., Chadefaux, T., Donnay, K., Blanke, U., Woolley-Meza, O., Moussaid, M., Johansson, A., Krause, J., Schutte, S., and Perc, M. (2015). Saving human lives: what complexity science and information systems can contribute. J. Stat. Phys. *158*, 735–781. https://doi.org/10.1007/s10955-014-1024-9.
15. Sunohara, S., Asakura, T., Kimura, T., Ozawa, S., Oshima, S., Yamauchi, D., and Tamakoshi, A. (2021). Effective vaccine allocation strategies, balancing economy with infection control against COVID-19 in Japan. PLoS One *16*, e0257107. https://doi.org/10.1371/journal.pone.0257107.
16. Tuite, A.R., Fisman, D.N., Kwong, J.C., and Greer, A.L. (2010). Optimal Pandemic Influenza Vaccine Allocation Strategies for the Canadian Population. PLoS One *5*, e10520. https://doi.org/10.1371/journal.pone.0010520.
17. Foy, B.H., Wahl, B., Mehta, K., Shet, A., Menon, G.I., and Britto, C. (2021). Comparing COVID-19 vaccine allocation strategies in India: a mathematical modelling study. Int. J. Infect. Dis. *103*, 431–438. https://doi.org/10.1016/j.ijid.2020.12.075.
18. Han, S., Cai, J., Yang, J., Zhang, J., Wu, Q., Zheng, W., Shi, H., Ajelli, M., Zhou, X.H., and Yu, H. (2021). Time-varying optimization of COVID-19 vaccine prioritization in the context of limited vaccination capacity. Nat. Commun. *12*, 4673. https://doi.org/10.1038/s41467-021-24872-5.
19. Bauer, S., Contreras, S., Dehning, J., Linden, M., Iftekhar, E., Mohr, S.B., Olivera-Nappa, A., and Priesemann, V. (2021). Relaxing restrictions at the pace of vaccination increases freedom and guards against further COVID-19 waves. PLoS Comput. Biol. *17*, e1009288. https://doi.org/10.1371/journal.pcbi.1009288.
20. Pollicott, M., Wang, H., and Weiss, H.H. (2012). Extracting the time-dependent transmission rate from infection data via solution of an inverse ODE problem. J. Biol. Dyn. *6*, 509–523. https://doi.org/10.1080/17513758.2011.645510.
21. Peters, J., Janzing, D., and Schölkopf, B. (2017). Elements of Causal Inference: Foundations and Learning Algorithms (The MIT Press).
22. Ministry of Health Israel. COVID-19 *Database*.
23. Tartof, S.Y., Slezak, J.M., Fischer, H., Hong, V., Ackerson, B.K., Ranasinghe, O.N., Frankland, T.B., Ogun, O.A., Zamparo, J.M., Gray, S., et al. (2021). Effectiveness of mRNA BNT162b2 COVID-19 vaccine up to 6 months in a large integrated health system in the USA: a retrospective cohort study. Lancet *398*, 1407–1416. https://doi.org/10.1016/S0140-6736(21)02183-8.
24. Pung, R., Mak, T.M., CMMID COVID-19 working group, Kucharski, A.J., and Lee, V.J. (2021). Serial intervals in SARS-CoV-2 B.1.617.2 variant cases. Lancet *398*, 837–838. https://doi.org/10.1016/S0140-6736(21)01697-4.
25. Hart, W.S., Miller, E., Andrews, N.J., Waight, P., Maini, P.K., Funk, S., and Thompson, R.N. (2022). Generation time of the alpha and delta SARS-CoV-2 variants: an epidemiological analysis. Lancet Infect. Dis. *22*, 603–610. https://doi.org/10.1016/S1473-3099(22)00001-9.
26. Jerusalem Post Staff (2020). Third coronavirus lockdown rules - everything you need to know. In The Jerusalem Post.
27. Katz, G.M., Born, K.B., Balicer, R.D., Salmon, A., Barrett, K., Bell, C.M., Choi, Y., Desveaux, L., Evans, G.A., Hopkins, J., et al. (2021). Lessons learned from Israel's reopening during a nationwide COVID-19 vaccination campaign. Science Briefs of the Ontario COVID-19 Science Advisory Table *2*, 33. https://doi.org/10.47326/ocsat.2021.02.33.1.0.
28. Times of Israel Staff (2020). Israel reimposes indoor mask requirement as daily cases top 200. In The Times of Israel.
29. Labrisch, H. (2020). Green Pass in effect: Israelis require jab certificate for indoor events. In The Jerusalem Post.
30. Viboud, C., Eisenstein, J., Reid, A.H., Janczewski, T.A., Morens, D.M., and Taubenberger, J.K. (2013). Age-and sex-specific mortality associated with the 1918–1919 influenza pandemic in Kentucky. J. Infect. Dis. *207*, 721–729. https://doi.org/10.1093/infdis/jis745.
31. Vilches, T.N., Moghadas, S.M., Sah, P., Fitzpatrick, M.C., Shoukat, A., Pandey, A., and Galvani, A.P. (2022). Estimating COVID-19 infections,

hospitalizations, and deaths following the US vaccination campaigns during the pandemic. JAMA Netw. Open *5*, e2142725. https://doi.org/10.1001/jamanetworkopen.2021.42725.

32. Haas, E.J., McLaughlin, J.M., Khan, F., Angulo, F.J., Anis, E., Lipsitch, M., Singer, S.R., Mircus, G., Brooks, N., Smaja, M., et al. (2022). Infections, hospitalisations, and deaths averted via a nationwide vaccination campaign using the Pfizer–BioNTech BNT162b2 mRNA COVID-19 vaccine in Israel: a retrospective surveillance study. Lancet Infect. Dis. *22*, 357–366. https://doi.org/10.1016/S1473-3099(21)00566-1.
33. Bubar, K.M., Reinholt, K., Kissler, S.M., Lipsitch, M., Cobey, S., Grad, Y.H., and Larremore, D.B. (2021). Model-informed COVID-19 vaccine prioritization strategies by age and serostatus. Science *371*, 916–921. https://doi.org/10.1126/science.abe6959.
34. Matrajt, L., Eaton, J., Leung, T., and Brown, E.R. (2020). Vaccine optimization for COVID-19: who to vaccinate first? Sci. Adv. *7*, eabf1374. https://doi.org/10.1126/sciadv.abf1374.
35. Islam, M.R., Oraby, T., McCombs, A., Chowdhury, M.M., Al-Mamun, M., Tyshenko, M.G., and Kadelka, C. (2021). Evaluation of the United States COVID-19 vaccine allocation strategy. PLoS One *16*, e0259700. https://doi.org/10.1371/journal.pone.0259700.
36. Davies, N.G., Klepac, P., Liu, Y., Prem, K., Jit, M.; CMMID COVID-19 working group, and Eggo, R.M. (2020). Age-dependent effects in the transmission and control of COVID-19 epidemics. Nat. Med. *26*, 1205–1211. https://doi.org/10.1038/s41591-020-0962-9.
37. Stringhini, S., Wisniak, A., Piumatti, G., Azman, A.S., Lauer, S.A., Baysson, H., De Ridder, D., Petrovic, D., Schrempft, S., Marcus, K., et al. (2020). Seroprevalence of anti-SARS-CoV-2 IgG antibodies in Geneva, Switzerland (SEROCoV-POP): a population-based study. Lancet *396*, 313–319. https://doi.org/10.1016/S0140-6736(20)31304-0.
38. Mulberry, N., Tupper, P., Kirwin, E., McCabe, C., and Colijn, C. (2021). Vaccine rollout strategies: the case for vaccinating essential workers early. PLOS Glob. Public Health *1*, e0000020. https://doi.org/10.1371/journal.pgph.0000020.
39. Crook, H., Raza, S., Nowell, J., Young, M., and Edison, P. (2021). Long covid—mechanisms, risk factors, and management. BMJ *374*, n1648. https://doi.org/10.1136/bmj.n1648.
40. Nord, E. (1999). Cost-Value Analysis in Health Care: Making Sense Out of QALYS (Cambridge University Press). https://doi.org/10.1017/CBO9780511609145.
41. Dönges, P., Wagner, J., Contreras, S., Iftekhar, E.N., Bauer, S., Mohr, S.B., Dehning, J., Calero Valdez, A., Kretzschmar, M., Mäs, M., et al. (2022). Interplay between risk perception, behavior, and COVID-19 spread. Front. Phys. *10*. https://doi.org/10.3389/fphy.2022.842180.
42. Sergio, H., Mejia, G., Kirschbaum, E., and Janzing, D. (2022). Obtaining causal information by merging datasets with MAXENT. In International Conference on Artificial Intelligence and Statistics, pp. 581–603.
43. Gresele, L., Von Kügelgen, J., Kübler, J., Kirschbaum, E., Schölkopf, B., and Janzing, D. (2022). Causal inference through the structural causal marginal problem. In International Conference on Machine Learning, pp. 7793–7824.
44. Schölkopf, B., Locatello, F., Bauer, S., Ke, N.R., Kalchbrenner, N., Goyal, A., and Bengio, Y. (2021). Toward causal representation learning. Proc. IEEE *109*, 612–634. https://doi.org/10.1109/JPROC.2021.3058954.
45. United Nations; Department of Economic and Social Affairs; Population Division (2019). World Population Prospects 2019: Data Booklet. https://doi.org/10.18356/3e9d869f-en.
46. Kekić, A. (2023). covid-vaccine-evaluation. https://doi.org/10.5281/zenodo.7764466.
47. Fraser, C. (2007). Estimating individual and household reproduction numbers in an emerging epidemic. PLoS One *2*, e758. https://doi.org/10.1371/journal.pone.0000758.
48. Jacquez, J.A., Simon, C.P., Koopman, J., Sattenspiel, L., and Perry, T. (1988). Modeling and analyzing HIV transmission: the effect of contact patterns. Math. Biosci. *92*, 119–199. https://doi.org/10.1016/0025-5564(88)90031-4.
49. Feng, Z., Hill, A.N., Smith, P.J., and Glasser, J.W. (2015). An elaboration of theory about preventing outbreaks in homogeneous populations to include heterogeneity or preferential mixing. J. Theor. Biol. *386*, 177–187. https://doi.org/10.1016/j.jtbi.2015.09.006.
50. Mistry, D., Litvinova, M., Pastore Y Piontti, A., Chinazzi, M., Fumanelli, L., Gomes, M.F.C., Haque, S.A., Liu, Q.H., Mu, K., Xiong, X., et al. (2021). Inferring high-resolution human mixing patterns for disease modeling. Nat. Commun. *12*, 323. https://doi.org/10.1038/s41467-020-20544-y.
51. Prem, K., Zandvoort, K.v., Klepac, P., Eggo, R.M., Davies, N.G., Centre for the Mathematical Modelling of Infectious Diseases COVID-19 Working Group, Cook, A.R., and Jit, M. (2021). Projecting contact matrices in 177 geographical regions: an update and comparison with empirical data for the COVID-19 era. PLoS Comput. Biol. *17*, e1009098. https://doi.org/10.1371/journal.pcbi.1009098.
52. Salvatier, J., Wiecki, T.V., and Fonnesbeck, C. (2016). Probabilistic programming in Python using PyMC3. PeerJ Comput. Sci. *2*, e55. https://doi.org/10.7717/peerj-cs.55.
53. Hoffman, M.D., and Gelman, A. (2014). The No-U-turn sampler: adaptively setting path lengths in Hamiltonian Monte Carlo. J. Mach. Learn. Res. *15*, 1593–1623. https://doi.org/10.5555/2627435.2638586.

Patterns, Volume *4*

Supplemental information

Evaluating vaccine allocation strategies using simulation-assisted causal modeling

Armin Kekić, Jonas Dehning, Luigi Gresele, Julius von Kügelgen, Viola Priesemann, and Bernhard Schölkopf

Supplemental Information

A. Supplemental Experimental Procedures

A.1. Generating vaccine allocation strategies

A.1.1. Factual strategy

In the available dataset [1] the vaccination times for first, second and third doses are given for each age group, *i.e.*

$$P(T_i|A) \quad \text{for } i = 1, 2, 3. \tag{43}$$

However, this alone does not uniquely determine the distribution of waning times. To fully specify required joint distributions $P(T_1, T_2|A)$ and $P(T_3|T_2, A)$ we employ a greedy algorithm to

1. Maximise the number of vaccine recipients that receive their second dose 3 weeks after their initial dose (or as close to 3 weeks as possible, but not sooner).
2. Have a minimum gap of 12 weeks between second and third dose (constraint).
3. Satisfy (43) (constraint).

A.1.2. Uniform strategy

For all counterfactual strategies we require the number of first, second and third doses to match the factual—but not in each age group. In other words,

$$\tilde{P}(T_i) = P(T_i) \quad \text{for } i = 1, 2, 3. \tag{44}$$

where the tilde indicates probabilities in the counterfactual scenario. For the `Uniform` strategy, we require all age groups to have the same vaccination time distributions:

$$\tilde{P}(T_1, T_2|A) = \tilde{P}(T_1, T_2) \quad \text{and} \quad \tilde{P}(T_3|T_2, A) = \tilde{P}(T_3|T_2). \tag{45}$$

In summary, the greedy algorithm to determine $\tilde{P}(T_1, T_2|A)$ and $\tilde{P}(T_3|T_2, A)$ is adapted as follows:

1. Maximise the number of vaccine recipients that receive their second dose 3 weeks after their initial dose (or as close to 3 weeks as possible, but not sooner).
2. Have a minimum gap of 12 weeks between second and third dose (constraint).
3. Satisfy (44) (constraint).
4. Satisfy (45) (constraint).

A.1.3. Ranked strategies

In the ranked strategies `ElderlyFirst`, `YoungFirst`, `RiskRanked` and `RiskRankedReversed` all age groups are ranked and vaccinations are assigned to the highest-ranked age group its vaccine uptake rate is reached. The counterfactual vaccine uptake rate per age group is the factual one for the first and second doses, *i.e.*

$$\sum_{t_i=1}^{M} \tilde{P}(t_i|a) \leq \sum_{t_i=1}^{M} P(t_i|a) \quad \text{for } i = 1, 2 \text{ and } \forall a. \tag{46}$$

The vaccine uptake rate for booster shots is slightly relaxed by 2.5% to meet other consistency constraints:

$$\sum_{t_3=1}^{M} \tilde{P}(t_3|a) \leq 0.025 + \sum_{t_3=1}^{M} P(t_3|a) \quad \forall a. \tag{47}$$

The greedy algorithm is adapted to

1. Prioritise age groups according to ranking.
2. Maximise the number of vaccine recipients that receive their second dose 3 weeks after their initial dose (or as close to 3 weeks as possible, but not sooner).
3. Have a minimum gap of 12 weeks between second and third dose (constraint).
4. Satisfy (44) (constraint).
5. Satisfy (46) and (47) (constraint).

A.2. Target function

Our goal is to compute the expected number of severe cases after intervening on the distribution of vaccination times:

$$s(\tilde{\pi} = \tilde{P}(T_1, T_2, T_3|A)) := MD\, \mathbb{E}[S|\text{do}(T_1, T_2, T_3 \sim \tilde{P}(T_1, T_2, T_3|A))] \tag{48}$$

where $\tilde{P}(T_1, T_2, T_3|A)$ is the distribution of vaccination times in the counterfactual scenario. Using results for stochastic policies from [2], repeated application of the law of total probabilities (TP) and exploiting conditional independences (CI) implied by the causal graph (see Figure 2) we can derive an expression for (48) in terms of known conditional probabilities:

$$\begin{aligned}
&s(\tilde{\pi} = \tilde{P}(T_1, T_2, T_3|A)) \\
&:= MD\ \mathbb{E}[S|\text{do}(T_1, T_2, T_3 \sim \tilde{P}(T_1, T_2, T_3|A))] && (49)\\
&= MD\ P\left(S{=}1|\text{do}(T_1, T_2, T_3 \sim \tilde{P}(T_1, T_2, T_3|A))\right) && (50)
\end{aligned}$$

$$\stackrel{[2, \text{Ch. } 4.2]}{=} MD \sum_a P(a) \sum_{t_1,t_2,t_3=1}^{M+1} \tilde{P}(t_1, t_2, t_3|a)$$

$$P(S{=}1|\text{do}(t_1, t_2, t_3), a) \tag{51}$$

$$\stackrel{\text{TP}}{=} MD \sum_a P(a) \sum_{t_1,t_2,t_3=1}^{M+1} \tilde{P}(t_1, t_2, t_3|a) \sum_{t=1}^{M} \underbrace{P(t|\text{do}(t_1, t_2, t_3), a)}_{\stackrel{\text{CI}}{=} P(t)}$$

$$P(S{=}1|\text{do}(t_1, t_2, t_3), a, t) \tag{52}$$

$$\stackrel{\text{TP}}{=} MD \sum_a P(a) \sum_{t=1}^{M} \underbrace{P(t)}_{=1/M} \sum_{t_1,t_2,t_3=1}^{M+1} \tilde{P}(t_1, t_2, t_3|a)$$

$$\sum_{w=1}^{M} \underbrace{P(w|\text{do}(t_1, t_2, t_3), a, t)}_{\stackrel{\text{CI}}{=} P(w|\text{do}(t_1,t_2,t_3),t)} P(S{=}1|\text{do}(t_1, t_2, t_3), a, t, w) \tag{53}$$

$$\stackrel{\text{TP}}{=} D \sum_a P(a) \sum_{t=1}^{M} \sum_{t_1,t_2,t_3=1}^{M+1} \tilde{P}(t_1, t_2, t_3|a) \sum_{w=1}^{M} P(w|\text{do}(t_1, t_2, t_3), t)$$

$$\sum_{v=0}^{3} \underbrace{P(v|\text{do}(t_1, t_2, t_3), a, t, w)}_{\stackrel{\text{CI}}{=} P(v|\text{do}(t_1,t_2,t_3),t)} \underbrace{P(S{=}1|\text{do}(t_1, t_2, t_3), v, a, t, w)}_{\stackrel{\text{CI}}{=} P(S=1|v,a,t,w)} \tag{54}$$

$$= D \sum_a P(a) \sum_{t=1}^{M} \sum_{t_1,t_2,t_3=1}^{M+1} \tilde{P}(t_1, t_2, t_3|a) \sum_{w=1}^{M} P(w|\text{do}(t_1, t_2, t_3), t)$$

$$\sum_{v=0}^{3} P(v|\text{do}(t_1, t_2, t_3), t) \; P(S{=}1|v, a, t, w) \tag{55}$$

$$= D \sum_a P(a) \sum_{t=1}^{M} \sum_{t_1,t_2,t_3=1}^{M+1} \tilde{P}(t_1, t_2, t_3|a) \sum_{w=1}^{M} P(w|t_1, t_2, t_3, t)$$

$$\sum_{v=0}^{3} P(v|t_1, t_2, t_3, t) \; P(S{=}1|v, a, t, w). \tag{56}$$

In the last step we use that since $\{T_1, T_2, T_3, T\}$ are the parents of V and W we can replace the do-operators by conditional probabilities [2]. Also note that for notational convenience, we set the vaccination time $t_i = M + 1$ for a patient who has not received the ith dose during the considered time window $t \in \{1, \ldots, M\}$.

The waning time w (number of weeks since the last dose was received) depends deter-

ministically on the vaccination times t_1, t_2, t_3:

$$P(w|t_1,t_2,t_3,t) = \begin{cases} 1 & \text{if } w = \max_{i\in\{1,2,3\}}[t-t_i]^+, \\ 0 & \text{else,} \end{cases}$$
$$\text{where } [x]^+ = \begin{cases} x & \text{if } x \geq 0, \\ 0 & \text{else.} \end{cases} \tag{57}$$

The vaccination status v also depends deterministically on the vaccination times t_1, t_2, t_3:

$$P(v|t_1,t_2,t_3,t) = \begin{cases} 1 & \text{if } v = \max_{i\in\{0,1,2,3\}}\left(i \cdot \operatorname{sgn}(t-t_i+1)\right), \\ 0 & \text{else,} \end{cases} \tag{58}$$

where sgn is the sign function. These deterministic relationships can be used to eliminate the corresponding conditionals from the target function:

$$\begin{aligned} & s(\tilde{\pi} = \tilde{P}(T_1,T_2,T_3|A)) \\ & = D \sum_a P(a) \sum_{t=1}^{M} \\ & \times \Bigg[\sum_{t_1=t+1}^{M+1} \sum_{t_2=t+1}^{M+1} \sum_{t_3=t+1}^{M+1} \tilde{P}(t_1,t_2,t_3|a)\ P(S{=}1|t,a,v{=}0,w{=}0) \\ & + \sum_{t_1=1}^{t} \sum_{t_2=t+1}^{M+1} \sum_{t_3=t+1}^{M+1} \tilde{P}(t_1,t_2,t_3|a)\ P(S{=}1|t,a,v{=}1,w{=}(t-t_1)) \\ & + \sum_{t_1=1}^{t} \sum_{t_2=1}^{t} \sum_{t_3=t+1}^{M+1} \tilde{P}(t_1,t_2,t_3|a)\ P(S{=}1|t,a,v{=}2,w{=}(t-t_2)) \\ & + \sum_{t_1=1}^{t} \sum_{t_2=1}^{t} \sum_{t_3=1}^{t} \tilde{P}(t_1,t_2,t_3|a)\ P(S{=}1|t,a,v{=}3,w{=}(t-t_3)) \Bigg]. \end{aligned} \tag{59}$$

Substituting the factorisation for the severity mechanism $P(S{=}1|v,a,t,w)$ we get:

$$
\begin{aligned}
&s(\tilde{\pi} = \tilde{P}(T_1, T_2, T_3|A)) \\
&= D \sum_{a} P(a) \sum_{t=1}^{M} f^0(t) f^1_{\tilde{\pi}}(a,t) \\
&\quad \times \Bigg[\sum_{t_1=t+1}^{M+1} \sum_{t_2=t+1}^{M+1} \sum_{t_3=t+1}^{M+1} \tilde{P}(t_1,t_2,t_3|a)\; g(0,a) \\
&\quad + \sum_{t_1=1}^{t} \sum_{t_2=t+1}^{M+1} \sum_{t_3=t+1}^{M+1} \tilde{P}(t_1,t_2,t_3|a)\; g(1,a)\, h^1(t-t_1) \\
&\quad + \sum_{t_1=1}^{t} \sum_{t_2=1}^{t} \sum_{t_3=t+1}^{M+1} \tilde{P}(t_1,t_2,t_3|a)\; g(2,a)\, h^2(t-t_2) \\
&\quad + \sum_{t_1=1}^{t} \sum_{t_2=1}^{t} \sum_{t_3=1}^{t} \tilde{P}(t_1,t_2,t_3|a)\; g(3,a)\, h^3(t-t_3) \Bigg] .
\end{aligned} \tag{60}
$$

B. Base reproduction number estimates

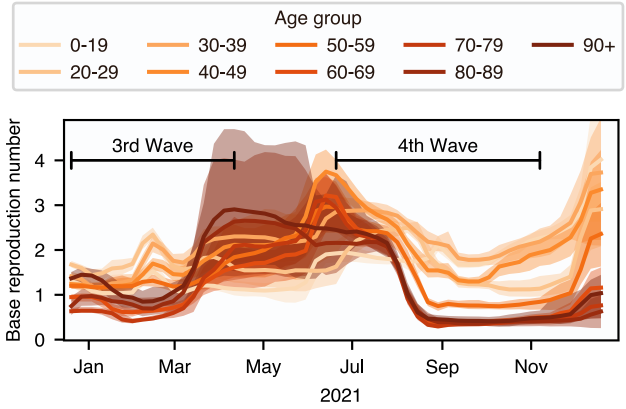

Figure S1: **Base reproduction numbers per age group inferred by the SEIR-like infection dynamics model.** The base reproduction numbers describe the contribution of each age group to infection spread after accounting for the effect of vaccination. The plot shows inferred weekly base reproduction numbers between 2020-12-20 and 2021-12-25. The shaded areas show the 95% credible intervals. The periods with high uncertainty coincide with low-incidence periods.

C. Results assuming other mixing factors

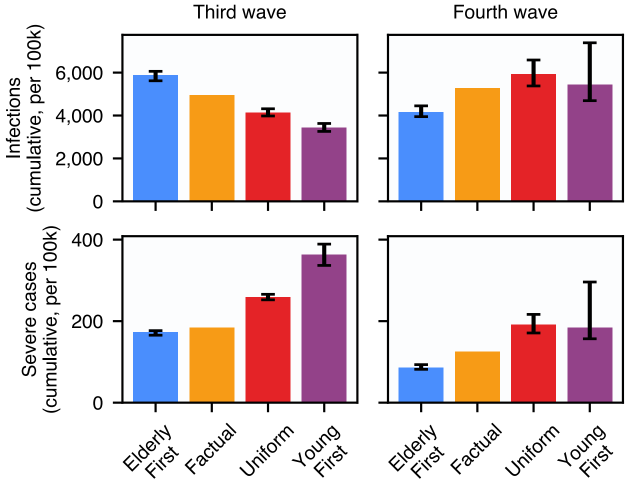

Figure S2: **Cumulative incidences of infections (top row) and severe cases (bottom row) for the two infection waves in 2021 under the factual and counterfactual vaccine allocation strategies, assuming a contact mixing factor of** 0.7**.** For the third wave we sum all cases from 2020-12-20 to 2021-04-11; for the fourth wave from 2021-06-20 to 2021-11-07. The whiskers show the 95% credible intervals.

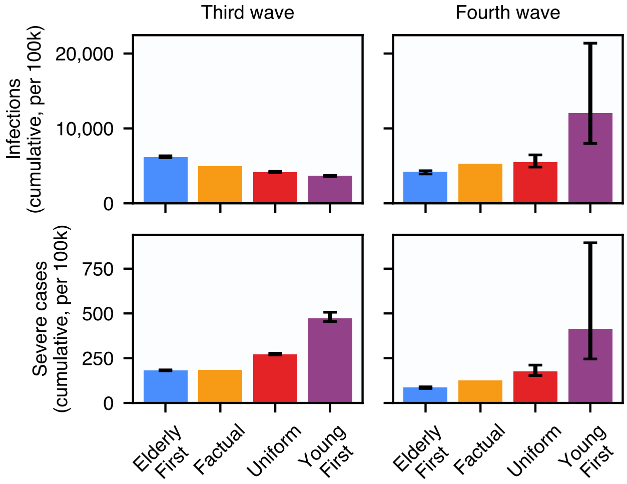

Figure S3: **Cumulative incidences of infections (top row) and severe cases (bottom row) for the two infection waves in 2021 under the factual and counterfactual vaccine allocation strategies, assuming a contact mixing factor of** 0.9**.** For the third wave we sum all cases from 2020-12-20 to 2021-04-11; for the fourth wave from 2021-06-20 to 2021-11-07. The whiskers show the 95% credible intervals.

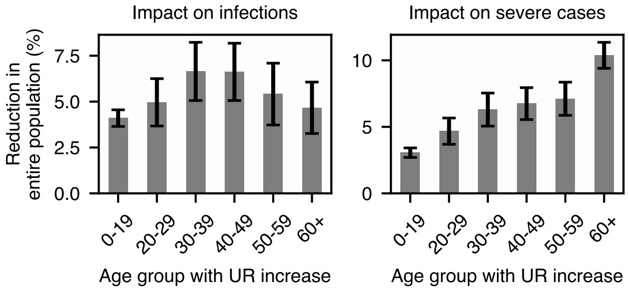

Figure S4: **Impact of increasing vaccine uptake rate (UR) in a given age group on severe cases, assuming a contact mixing factor of** 0.7**.** In each scenario, the vaccine uptake rate is increased in a given age group by a fixed number corresponding to 0.6% of the population being motivated to get vaccinated. We assume that the change comes from originally unvaccinated individuals who are persuaded to receive three doses. The plots show the impact on cumulative infections (left) and severe cases (right) in the entire population—not just in the age group in which the UR was increased. We consider cases from 2020-12-20 to 2021-12-25. The whiskers show the 95% credible intervals.

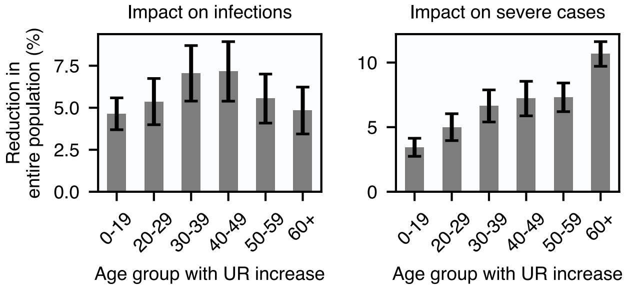

Figure S5: **Impact of increasing vaccine uptake rate (UR) in a given age group on severe cases, assuming a contact mixing factor of** 0.9**.** In each scenario, the vaccine uptake rate is increased in a given age group by a fixed number corresponding to 0.6% of the population being motivated to get vaccinated. We assume that the change comes from originally unvaccinated individuals who are persuaded to receive three doses. The plots show the impact on cumulative infections (left) and severe cases (right) in the entire population—not just in the age group in which the UR was increased. We consider cases from 2020-12-20 to 2021-12-25. The whiskers show the 95% credible intervals.

References

[1] Ministry of Health Israel. *COVID-19 Database*.

[2] Judea Pearl. *Causality*. Cambridge University Press, 2009. DOI: https://doi.org/10.1017/CBO9780511803161.
